# Overexpression of MFN2 alleviates sorafenib-induced cardiomyocyte necroptosis via the MAM-CaMKIIδ pathway *in vitro* and *in vivo*

**DOI:** 10.7150/thno.65716

**Published:** 2022-01-01

**Authors:** Ziping Song, Haixu Song, Dan Liu, Bing Yan, Daowen Wang, Yan Zhang, Xiaojie Zhao, Xiaoxiang Tian, Chenghui Yan, Yaling Han

**Affiliations:** 1 Division of Cardiology, Department of Internal Medicine, Tongji Hospital, Tongji Medical College, Huazhong University of Science and Technology, Wuhan 430030, China; 2 Department of Cardiology and Cardiovascular Research Institute of PLA, General Hospital of Northern Theater Command, Shenyang 110016, China.

**Keywords:** Cardio-Oncology, Sorafenib, MFN2, Necroptosis, MAM

## Abstract

**Background:** The continued success of oncological therapeutics is dependent on the mitigation of treatment-related adverse events, particularly cardiovascular toxicities. As such, there is an important need to understand the basic mechanisms of drug toxicities in the process of antitumor therapy. Our aim in this study was to elucidate the underlying mechanisms of sorafenib (sor)-induced cardiomyocyte damage.

**Methods:** Primary mouse cardiomyocytes were prepared and treated with sor and various other treatments. Cardiomyocyte necroptosis was detected by flow cytometry, western blotting, and CCK8 assays. Mitochondrial Ca^2+^ uptake was detected by the Rhod-2 probe using confocal imaging. Morphological changes in mitochondria and mitochondria-associated endoplasmic reticulum (ER) membranes (MAMs) were imaged using transmission electron microscopy (TEM) and confocal microscopy. Cardiac perfusion was performed to detect cardiac specific role of MFN2 overexpression *in vivo*.

**Results:** We reported that mitochondrial Ca^2+^ overload, the subsequent increase in calmodulin-dependent protein kinase II delta (CaMKIIδ) and RIP3/MLKL cascade activation, contributed to sor-induced cardiac necroptosis. Excess MAM formation and close ER-mitochondria contact were key pathogenesis of sor-induced Ca^2+^ overload. Sor mediated MFN2 downregulation in a concentration-dependent manner. Furthermore, we found that reduced mitofusin-2 (MFN2) level augmented sor-mediated elevated MAM biogenesis and increased mitochondria-MAM tethering in cardiomyocytes. Sor-induced Mammalian Target of Rapamycin (mTOR) inactivation, followed by the activation and nuclear translocation of Transcription Factor EB (TFEB), contributed to mitophagy and MFN2 degradation. In an* in vivo* model, mice subjected to sor administration developed cardiac dysfunction, autophagy activation and necroptosis; our investigation found that global and cardiac-specific overexpression of MFN2 repressed cardiac dysfunction, and sor-induced cardiomyocyte necroptosis via repressing the MAM-CaMKIIδ-RIP3/MLKL pathway.

**Conclusion**: Sorafenib mediated cardiomyocyte necroptosis through the MFN2-MAM-Ca^2+^-CaMKIIδ pathway *in vitro* and *in vivo*. The overexpression of MFN2 could rescue sor-induced cardiomyocyte necroptosis without disturbing the anti-tumor effects.

## Introduction

In recent years, the advancement of anti-tumor medicine and therapy technology has greatly improved the survival rate and treatment quality of cancer patients. However, cardiac complications caused by anti-tumor drugs are still common and inevitable 1, 2. Tyrosine kinase inhibitors TKIs, due to their multitarget inhibitory effects on the vascular endothelial growth factor (VEGF) and mitogen-activated protein kinase (MAPK) pathways, have been extensively used as clinical first-line drugs for hepatocellular carcinoma (HCC), renal cell carcinoma 3, etc. Sorafenib (sor), a key and representative TKI, has improved the prognosis of cancer patients and has been especially recommended for advanced hepatocellular carcinoma 4, 5. However, it causes many cardiovascular complications, such as QT prolongation, heart failure, myocardial infarction, and hypertension 6. In a randomized double-blind phase-III trial, sor was found to induce more myocardial ischemic events than the placebo did 7. In addition, another study reported that sor elevated mouse mortality after myocardial infarction, as it mediated necrosis in cardiomyocytes by inhibiting the growth of bone marrow-derived stem cells and the activity of mitochondrial complexes II, III, V 8. However, the specific cardiomyocyte death pathway mediated by sor and the underlying mechanisms have not been fully elucidated.

Programmed cell necrosis, necroptosis, is a form of programmed cell death that differs from apoptosis and traditional necrosis. It is usually initiated by the tumor necrosis factor receptor (TNFR), Fas ligand receptor (FasR), and toll-like receptors (TLRs). In the absence of caspase 8, necroptosis is triggered by a signaling cascade composed of receptor-interacting serine/threonine-protein kinase 1 (RIPK1), receptor-interacting serine/threonine-protein kinase (RIPK3), and mixed lineage kinase domain-like protein (MLKL). Cascade phosphorylation of RIP1/RIP3/MLKL contributes to classical necroptosis. In addition to classical necroptosis, excessive opening of mitochondrial permeability transition pores (mPTPs) and mitochondrial-specific necroptosis boosted by Cyclophilin D (CypD) also play pivotal roles in cell death. After cells undergo necroptosis, their damage-associated molecular patterns (DAMPs) are exposed to the outside, and then eliminated by phagocytes 9, 10. Recently, some studies have reported that tumor cells are prone to apoptosis resistance and escape; therefore, promoting the necroptosis of tumor cells has become a new anti-tumor therapy strategy 11. Moreover, previous studies have reported that sor exerts anti-tumor effects by mediating necroptosis in tumor cells 12. However, it is still unclear whether sor stimulates cardiomyocyte necroptosis, which can contribute to cardiac damage. Thus, a major goal in the field is finding potential molecules that can maintain anti-tumor activity and protect against cardiovascular complications caused by anti-tumor medicines.

Mitofusin-2 (MFN2), a member of the mitochondrial fusion protein family located in the outer mitochondrial membrane, has already been reported to possess anti-tumor potential via various mechanisms, such as inducing apoptosis and promoting promoter DNA hypermethylation 13-16. Previous evidence has demonstrated that MFN2 is downregulated in hepatoma cells of HCC patients. Overexpression of MFN2 can effectively promote cancer cell apoptosis 17. Another study confirmed that patients with lower MFN2 expression always have a poor prognosis 18. Furthermore, MFN2 is located in the mitochondria-associated endoplasmic reticulum (ER) membrane (MAM), where it regulates Ca^2+^ transport from the ER to the mitochondria 19, 20. In addition, MFN2 downregulation induces excessive coupling of mitochondria and ER, triggering excess mitochondrial Ca^2+^ entry in cardiomyocytes 21. However, whether MFN2 regulates sor-induced cardiomyocyte toxicity by modulating Ca^2+^ homeostasis, as well as the underlying mechanisms of this process, are still unclear.

In the present study, we investigated how sor triggered necroptosis by disrupting Ca^2+^ homeostasis and how excess MFN2 degradation due to excessive mitophagy facilitated sor induced necroptosis in cardiomyocytes. Our results revealed that MFN2 could serve as novel intervention targets for future pharmacological therapies against TKI-induced cardiac complications.

## Methods

### Cell culture

Primary mouse cardiomyocytes were prepared from neonatal mice (days 0-2), digested with 0.2% collagenase II, neutralized with two times the volume of serum, and filtered with a 100 μm screen. Cardiomyocytes were then cultured in H-DMEM supplemented with 20% FBS, penicillin (100 U/mL), and streptomycin (100 μg/mL) at 37°C with 5% CO_2_ and 95% air. HL-1 cells were purchased from ATCC (Manassas, VA). The cells were then divided into different groups according to the experiment, as follows: adenovirus-loaded GFP or MFN2 (OBiO Technology (Shanghai) Corp., Ltd., Shanghai, China), KN93 (HY-15465, MCE, NJ), si-NC, si-FUNDC1, and si-MFN2 (RIBOBIO, Guangzhou, China), with or without sor (HY-10201, MCE).

### Animal experiments

All animal care and experimental procedures were approved by the Cardiovascular Research Institute and the Department of Cardiology of the General Hospital of Northern Theater Command for compliance with the National Institutes of Health for use of laboratory animals or equivalent. Male C57BL/6 mice (at eight weeks) from the Model Animal Research Center of Nanjing University (Nanjing, China) were employed. Mice were provided with 12 h light/12 h dark photoperiods and free access to food and water. Adeno-associated virus carrying the CMV promoter from OBiO Technology (H17690, Shanghai, China) and AAV-TNT-GFP-MFN2 and control virus AAV-TNT-GFP from HanbioTech (HH20210824DY-AAV01, Shanghai, China) were injected into the tail of mice for three weeks, and followed that sorafenib (40 mg/kg/d) was administered by gavage for another two months. For evaluating Necrostatin-1 (Nec-1) induced cardiac function, Nec-1 (1.65 mg/kg/d) was administrated subcutaneously by osmotic pumps (DURECT Corporation, Alzet model 1004) for 1 week before sor application.

### Cardiac perfusion

LV cardiomyocytes are isolated enzymatically using a Langendroff perfusion system as previously described 22. Briefly, langendroff perfusion system was set up firstly, then mice were anesthetized and hearts was transferd to a 60-mm dish and wash it with NT solution. mount the aorta onto the Langendorff perfusion cannula, and then firmly ligate the aorta onto the cannula. Then switch the perfusate to 30 mL Ca^2+^ -free Tyrode solution (10 mM Taurine, 1 mg/mL BSA) to stop the heartbeat. Next, switch the perfusate to Collagenase A isolation solution (0.6 mg/mL type II collagenase) for enzyme digestion for 20 min, and then quickly change the perfusion solution to the Ca^2+^ -free Tyrode solution to stop further digestion. Finally, place the heart in a 35-mm dish containing KB solution and cut off the LV tissue into small pieces. Centrifuge at 150 x g for 30 s and discard the supernatant. Re-suspend the myocytes in 10 mL KB solution, free settle for 6 min, discard the supernatant, and re-suspend the pellet in KB solution for microscopic observation and IP lysis buffer for following western blotting.

### Echocardiography measurements

At the end of the animal experiments, mouse cardiac echocardiography measurements were performed under 1% isoflurane anesthesia to detect cardiac function, and mouse cardiac function was detected using a high-resolution imaging system with a 30-MHz high-frequency scan head (Visual SonicsVevo-2100, Visual Sonics Inc., Toronto, Canada) as previously described. Data was analyzed using Vevo Strain software (FUJIFILM VisualSonics).

### Gene silencing and adenovirus infection

Briefly, cardiomyocytes were transfected with si-MFN2, si-FUNDC1, si-LAMP2 (10 μM; RIBOBIO, Guangzhou, China) using RNAiMAX (#13778150, Life Technologies, Carlsbad, CA). For overexpression of MFN2, adenovirus encoding MFN2 (OBiO Technology (Shanghai) Corp., Ltd.) was used to transfect primary neonatal mouse cardiomyocytes and HL-1 cells.

### Flow cytometry analysis of cardiomyocytes necrotic proportion

First, cells were digested in a 6-well plate, centrifuged at 1000 rpm for 5 min, resuspended in binding buffer, labeled with Annexin V and PI probes (#556547, BD Bioscience, San Jose, CA), and analyzed using the FACS flow cytometer (BD, Franklin Lakes, NJ). Cells in the upper right quadrants (PI positive and Annexin V positive) were considered necrotic.

### Ca^2+^ detection

To detect mitochondrial Ca^2+^ influx, a specific dye for mitochondrial Ca^2+^ uptake, Rhod-2 (R1245MP, Invitrogen, Thermo Fisher Scientific Inc., Waltham, MA) was employed. MitoTracker™ Green FM (M7514, Invitrogen, Thermo Fisher Scientific Inc.) was used to co-stain the mitochondria. Briefly, a stock solution of Rhod-2 was dissolved in DMSO, and cells were incubated with Rhod-2 at a concentration of 5 μM at 37°C in the dark for 30 min. Cells were washed three times to remove excess or non-specific probes loaded in mitochondria. The cells were then observed using a confocal microscope (LSM800, Carl Zeiss Microscopy Ltd., Cambridge, MA). To evaluate mitochondrial Ca^2+^ influx, a Z-stack was used to observe multiple layers. The Pearson correlation coefficient was used to quantify the degree of mitochondrial Ca^2+^ influx using the Zeiss colocalization module in the ZEN software, under thresholding obtained from a single-labelled sample for each separate channel.

### CCK8 assays

For cardiomyocyte activity measurements, Cell Counting Kit 8 (CCK8) assays were employed. Cells in 96-well plates were stimulated with various treatments for 24 h. Later, the supernatant was removed and replaced with the CCK8 solution (C0038, Beyotime Biotechnology, Shanghai, China) for 30 min at 37°C, and then cell viability was detected using a microplate reader to measure the OD value at 450 nm. Each group was set in five duplicate wells and experiments were repeated three times.

### RNA extraction and Real-time PCR

Total RNA from heart tissues and cardiomyocytes was extracted using TRIzol (15596018, Thermo Fisher Scientific) and reverse transcribed with reverse transcriptase (18080400, Thermo Fisher). The mRNA level was quantified using SYB and real-time PCR was performed using the ABI 7300 PCR System (Thermo Fisher). *Gapdh* served as an internal control, and the results were analyzed using the 2^-ΔΔCt^ method. Real-time PCR was performed on at least three different experimental samples. Detailed primers are listed in Supplementary Table S1.

### Western blotting

First, protein lysates were centrifuged at 12,000 × g at 4°C for 15 min, prepared with loading buffer, and boiled for 5 min. Then, they were electrophoresed with 10% SDS and transferred onto polyvinylidene fluoride membranes (Merck, Millipore). After blocking with 5% defatted milk for 1 h, membranes were incubated with primary antibodies overnight at 4°C, and the detailed information for primary antibodies was listed in Table S2. Membranes were then incubated with horseradish peroxidase-conjugated secondary antibodies for 2 h at room temperature. Finally, western blot results were analyzed and processed using Gel-Pro software.

### Transmission electron microscopy (TEM)

First, fresh hearts removed from mice or freshly collected cardiomyocytes were perfused with an electron microscope fixing solution (4% paraformaldehyde and 1% glutaraldehyde in 0.1 mol/L sodium cacodylate buffer, pH 7.4, and 4% sucrose). Then, 2% osmium tetroxide and 0.8% potassium ferrocyanide in a 0.1 mol/L sodium cacodylate buffer were employed to pre-fix for 2 h and odium cacodylate buffer was used to wash three times. The samples were dehydrated using a density gradient of alcohol and acetone. Finally, the samples were cut into 60-80 nm ultrathin slices, dyed, and dried overnight at room temperature. The sections were imaged and analyzed using an H-7600 TEM (Hitachi High-Technologies Europe GmbH, Krefeld, Germany). The images were analyzed by Image J. For measurement of ER and Mitochondria contact, perimeter of ER was calculated. For MAM quantification, the ER perimeter connected to Mitochondria to total ER perimeter was calculated and normalized as previously instructed 23.

### ER and Mitochondria contact analysis

Cells were labeled with MitoTracker™ Deep Red FM (M22426, Invitrogen, Thermo Fisher Scientific Inc.) and ER-Tracker™ Green (E34251, Invitrogen, Thermo Fisher Scientific Inc.) at 37°C for 30 min, and observed using a confocal microscope (LSM800, Carl Zeiss Microscopy Ltd.). To evaluate ER and mitochondrial contact and co-localization, a Z-stack was applied to observe multiple layers. The Pearson correlation coefficient was applied to quantify the degree of colocalization of mitochondria and ER using the Zeiss colocalization module in the ZEN software, under thresholding obtained from a single-labelled sample for each respective channel.

### Statistical analysis

All data were analyzed using GraphPad Prism 8 software and are shown as the mean ± SEM. In our study, t-tests and one-way analyses of variance were employed to evaluate statistical significance. Statistical significance was set at P < 0.05.

## Results

### Sorafenib induced necroptosis in cardiomyocytes

**Figure 1:**
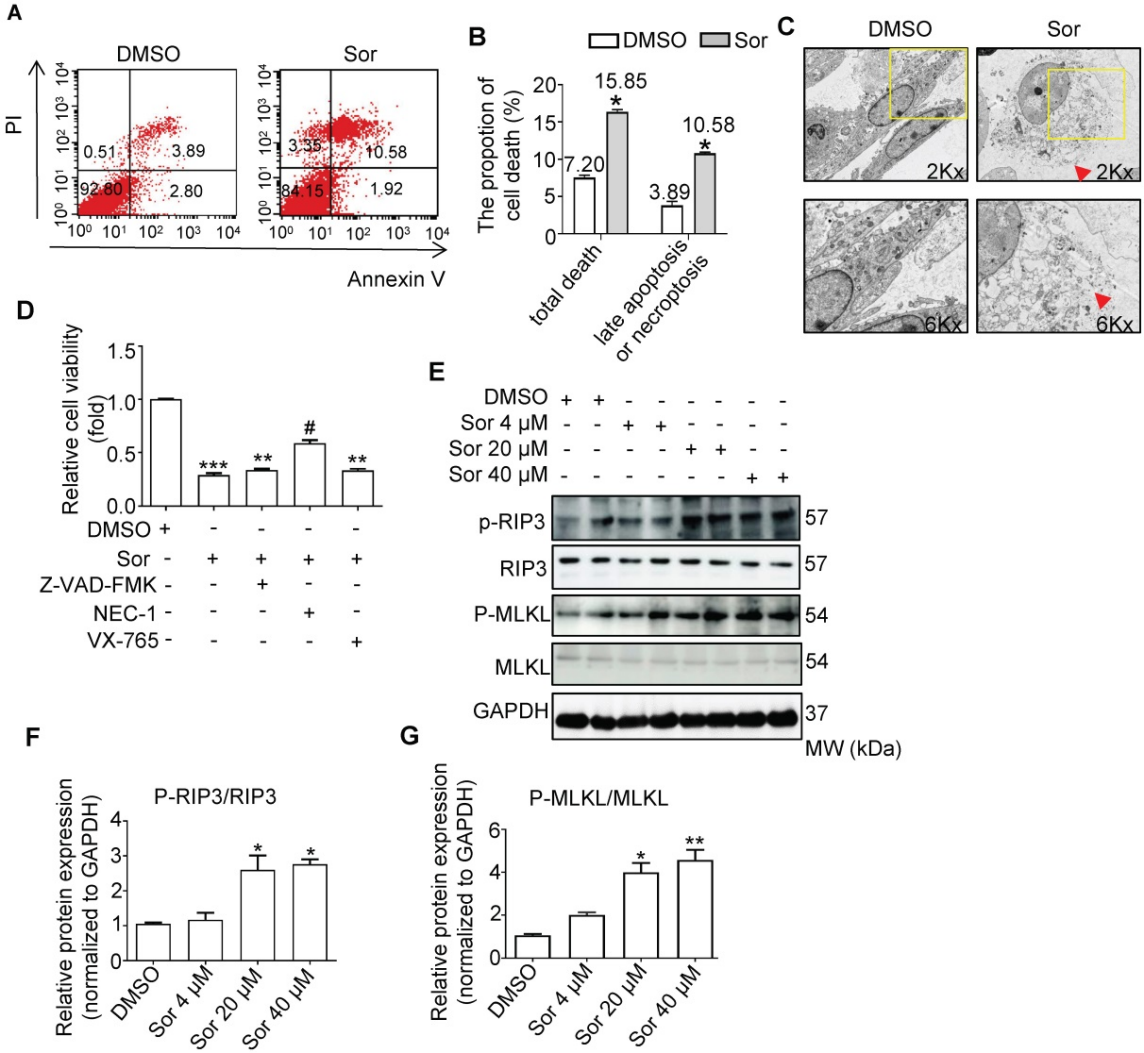
** Sorafenib-induced necroptosis in cardiomyocytes.** (A, B) Cardiomyocyte necrotic proportion under Sor application was detected by flow cytometry. (C) Necrotic morphology of cardiomyocytes imaged by transmission electron microscopy (TEM). The red arrows indicated cell rupture. (D) CCK8 assays were conducted to detect cell viability under various death inhibitors. Z-VAD-FMK as apoptosis inhibitor, 40 μM; NEC-1 as necroptosis inhibitor, 30 μM; VX-765 as pyroptosis inhibitor, 20 μM. (E-G) Representative blots and quantification of RIP3/MLKL pathway-induced necroptosis. Sor 20 μM was employed for experiments involved in Figure 1A-D. HL-1 cells were employed for experiments involved in Figure 1A, B, E, F, G. Primary neonatal mouse cardiomyocytes were employed for experiments involved in Figure 1C, D. ^*^P < 0.05 vs DMSO. ^**^P < 0.01 vs DMSO. ^#^*P* < 0.05 vs Sor 20 μM. N = 3.

As shown in Figure 1A and 1B, flow cytometry analysis revealed that sor induced cardiomyocyte death; late apoptosis or necroptosis (upper right quadrants) were upregulated by sor treatment. TEM analysis also confirmed that sor induced necrosis in cardiomyocytes (Figure 1C). To determine the exect cell death manner mediated by sor, various cell death inhibitors were employed to detect cell viability. As shown in Figure 1D, Z-VAD-FMK (as apoptosis inhibitor, 40 μM) or VX-765 (as pyroptosis inhibitor, 20 μM) application could not save cell viability. However, Necrostatin-1 (NEC-1 at 30 μM), a necroptosis inhibitor, blocked the sor-induced decrease of cell viability. Furthermore, classical necroptosis-related proteins, phosphorylated RIP3 and phosphorylated MLKL were detected to increase under sor stimulation in a dose-dependent manner, whereas RIP3 or MLKL was not changed by sor (Figure 1E-G).

### Mitochondrial Ca^2+^ overload mediated sor-induced necroptosis in cardiomyocytes

**Figure 2:**
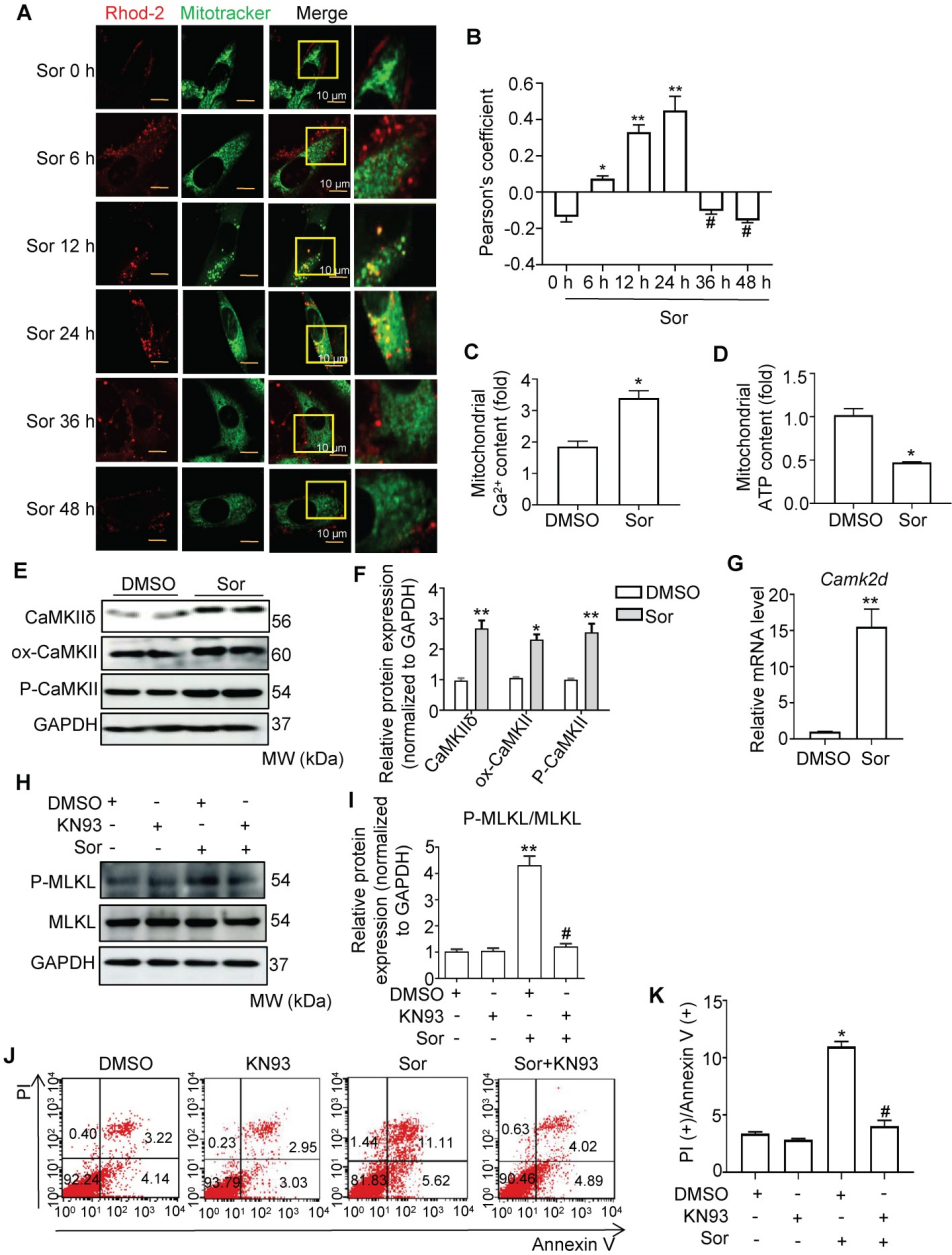
** Sorafenib triggered calcium overload to promote necroptosis.** (A, B) Representative confocal images and quantification of mitochondrial Ca2+ influx, labeled with Rhod-2 probe in a time-dependent manner. Scale bar: 10 μm. ^*^P < 0.05 vs Sor 0 h. ^**^P < 0.01 vs Sor 0 h. ^#^*P* < 0.05 vs Sor 24 h. (C, D) Isolated mitochondria in cardiomyocytes were detected for Ca2+ content and ATP level by kit. (E, F, G) Representative blots of CaMKIIδ and its two activation forms, and the relative mRNA level of CaMKIIδ. (H, I) Representative blots and analysis of proteins of interest stimulated with CaMKII inhibitor, KN93 (10 μM). (J, K) The necrotic proportion of cardiomyocytes stimulated with or without KN93 (10 μM) and Sor were detected by flow cytometry. All experiments used 20 μM of Sor. HL-1 cells were employed for experiments involved in Figure 2. Sor stimulation for 24 h was employed for Figure 2C-K. For Figure 2C-K, statistical significance was as follows: ^*^P < 0.05 vs DMSO. ^**^P < 0.01 vs DMSO. ^#^*P* < 0.05 vs Sor. N = 3.

Calcium overload in the mitochondria has been reported to be a key pathogenesis in necroptosis 24-27. Therefore, we investigated whether sor induced mitochondrial Ca^2+^ overload in cardiomyocytes. As shown in Figure 2A and 2B, Ca^2+^ influx in mitochondria first occurred at 6 h and reached a peak at 24 h after intervention with sor (20 μM). After 36 h of treatment with sor, mitochondrial Ca^2+^ influx was significantly reduced. In addition, mitochondrial Ca^2+^ content was increased by sor stimulation at 24 h. In contrast, ATP content decreased under sor stimulation at 24 h (Figure 2C-D). Due to the Ca^2+^ overload in the mitochondria, we further assessed CaMKIIδ (the main subunit in heart), one of the substrates of RIP3, which has been shown to be activated by Ca^2+^ in the mouse heart 28-31. Sor mediated CaMKIIδ elevated at the transcriptional level and CaMKII activation (marked by elevation of ox-CaMKII and P-CaMKII) (Figure 2E-G). The CaMKII inhibitor, KN93, effectively reversed sor-induced RIP3/MLKL pathway activation mediated necroptosis in cardiomyocytes investigated by western blotting and flow cytometry (Figure 2H-K).

### Sorafenib induced mitochondrial Ca^2+^ influx through upregulating of the calcium channel protein

To test whether mitochondrial Ca^2+^ overload in cardiomyocytes is derived from Ca^2+^ transport dysfunction, we detected the expression of mitochondrial Ca^2+^ uniporter (MCU), a pivotal mitochondrial Ca^2+^ entry protein, and mitochondrial Ca^2+^ export sodium-calcium exchanger (NCLX, encoded by the *SLC24A6* gene) 32-35. Western blotting showed rapid upregulation of MCU in cardiomyocytes after treatment with sor for 6 h. However, the NCLX protein level was not affected by sor treatment (Figure S1A-C). Real-time PCR demonstrated that sor induced the upregulation of *Mcu* at the mRNA level (Figure S1D). Silencing of MCU blocked sor-induced necroptosis by the RIP3/MLKL pathway (Figure S1E-F). Previously study has demonstrated CaMKII inhibition reduced MCU and is resistant to ischaemia reperfusion injury 36. Our result supported that KN93 inhibited *Mcu* expression at transcriptional level under sor stimulation (Figure S1G-I). In addition, silencing MCU via KN-93 employment reversed sor-induced decreased ATP content, and cell viability (Figure S1J-K).

### Mitochondria-associated ER membrane mediated sor-induced Ca^2+^ influx

**Figure 3:**
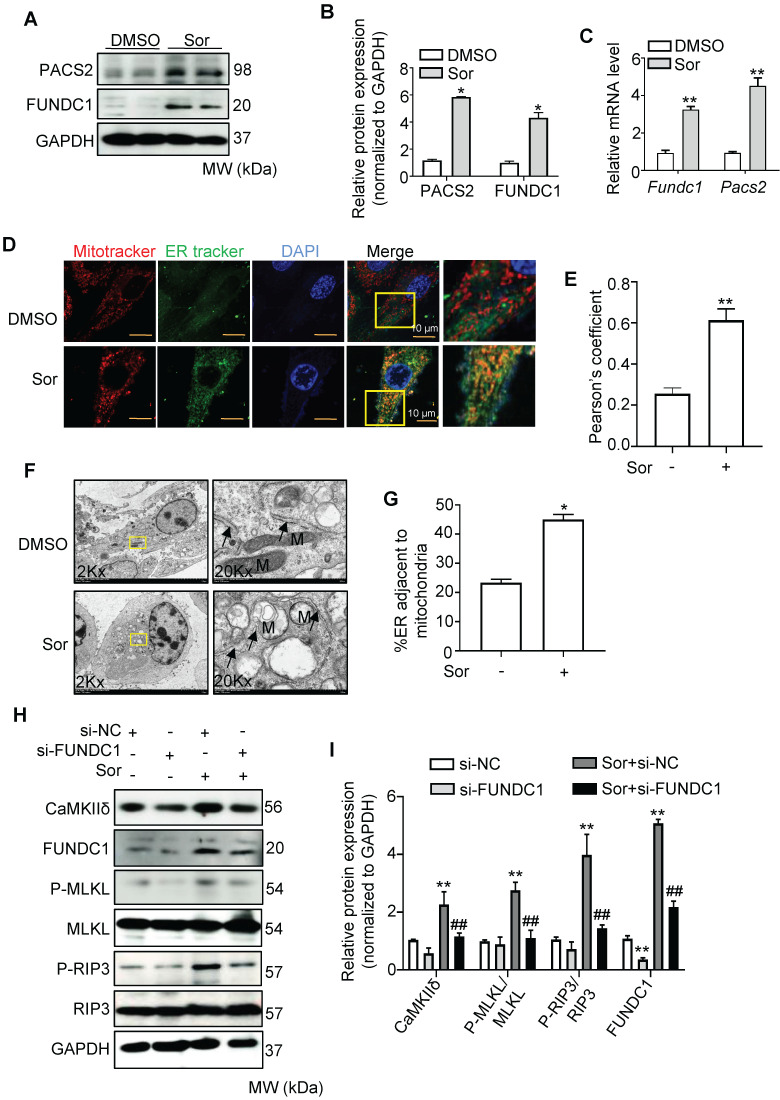
** Sor promoted ER-mito contact and upregulated CaMKII and Ca2+ overload-induced necroptosis.** (A, B) Representative blots and quantification of MAM marker proteins, PACS2 and FUNDC1. (C) Messenger RNA levels (mRNA) of MAM markers, fold change to Gapdh. (D, E) Representative staining of ER tracker and MitoTracker. The degree of colocalization of Mito and ER was assessed by Pearson's coefficient. Scale bar: 10 μm. (F, G) Representative images and quantification of sor-induced mitochondria-ER contact was detected by transmission electron microscopy. Arrows denote ER. M, mitochondria. (H, I) Representative blots and quantification of proteins of interest under FUNDC1 inhibition. HL-1 cells were employed for experiments involved in Figure 3D-G. Primary neonatal mouse cardiomyocytes were employed for experiments involved in other legends of Figure 3. ^*^P < 0.05 vs DMSO. ^**^P < 0.01 vs DMSO or si-NC. ^##^*P* < 0.01 vs Sor+si-NC. N = 3.

It has been reported that mitochondrial Ca^2+^ uptake from the cytosol requires direct contact between the mitochondria and the ER membrane via the MAM 37-39. Therefore, we investigated whether sor changed the MAM components. First, PACS2 and FUNDC1, two key components of MAMs, were detected to remarkably increase at both the mRNA and protein levels in cardiomyocytes after treatment with sor (Figure 3A-C). Second, confocal imaging and quantitative analysis indicated that Mito-ER contact was excessively close in cardiomyocytes with sor administration, compared to the untreated group (Figure 3D-E). TEM imaging also showed that exposure of cardiomyocytes to sor decreased the average distance between the ER and mitochondria (Figure 3F-G). These results implied that sor increased the biogenesis of MAMs and reduced the distance between MAMs and mitochondria. As expected, knockdown of FUNDC1 expression significantly inhibited sor-induced Ca^2+^ overflow in mitochondria (Figure S2A-B). Meanwhile, as shown in Figure 3H and 3I, silencing of FUNDC1 blocked sor-induced necroptosis and reversed CaMKIIδ expression, which indicated that changes in MAMs mediated Ca^2+^ overflow to increase CaMKIIδ expression and induce necroptosis.

### MFN2 is a key protein in regulating MAM biogenesis and the distance between mitochondria and MAMs

**Figure 4:**
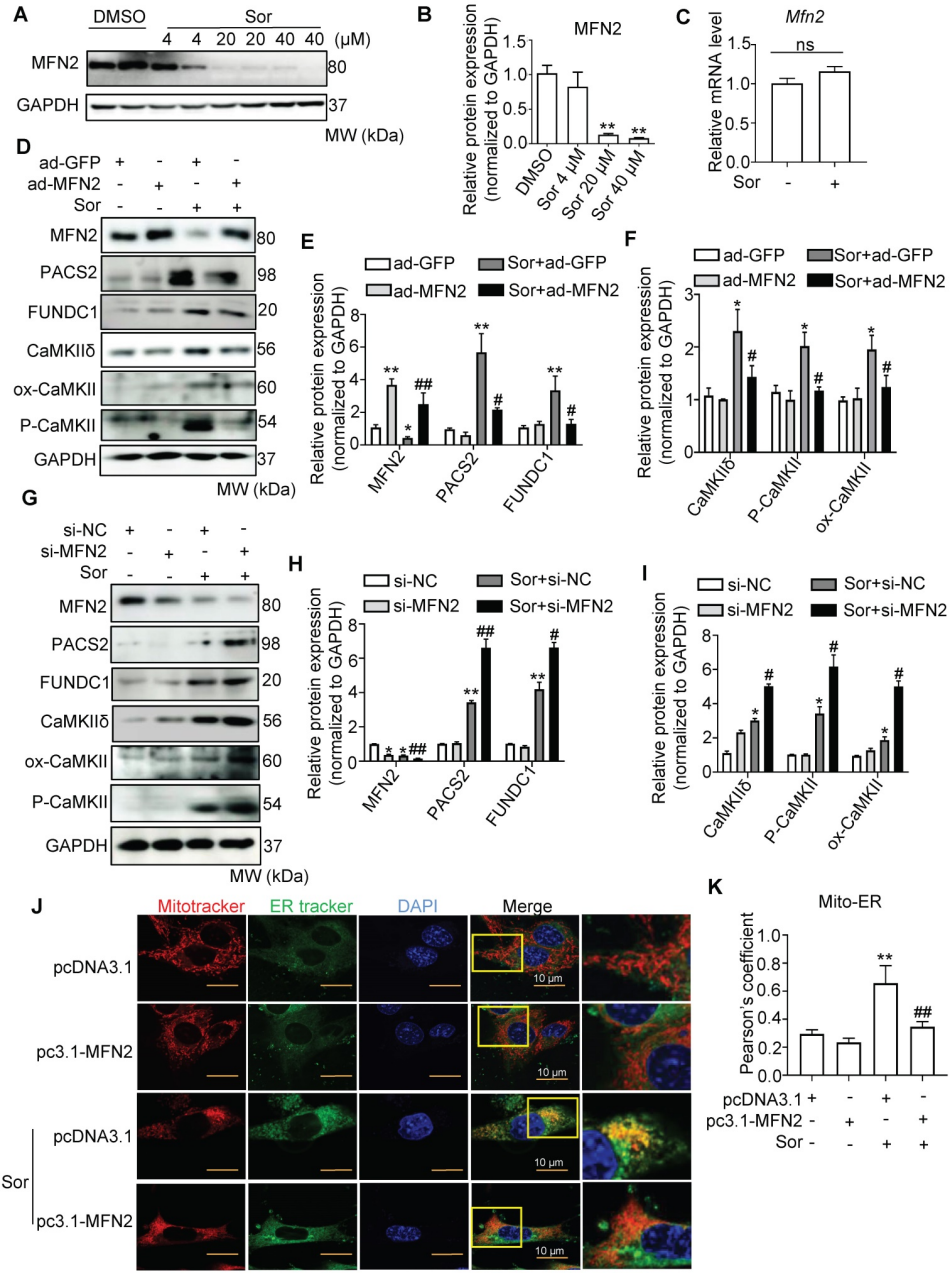

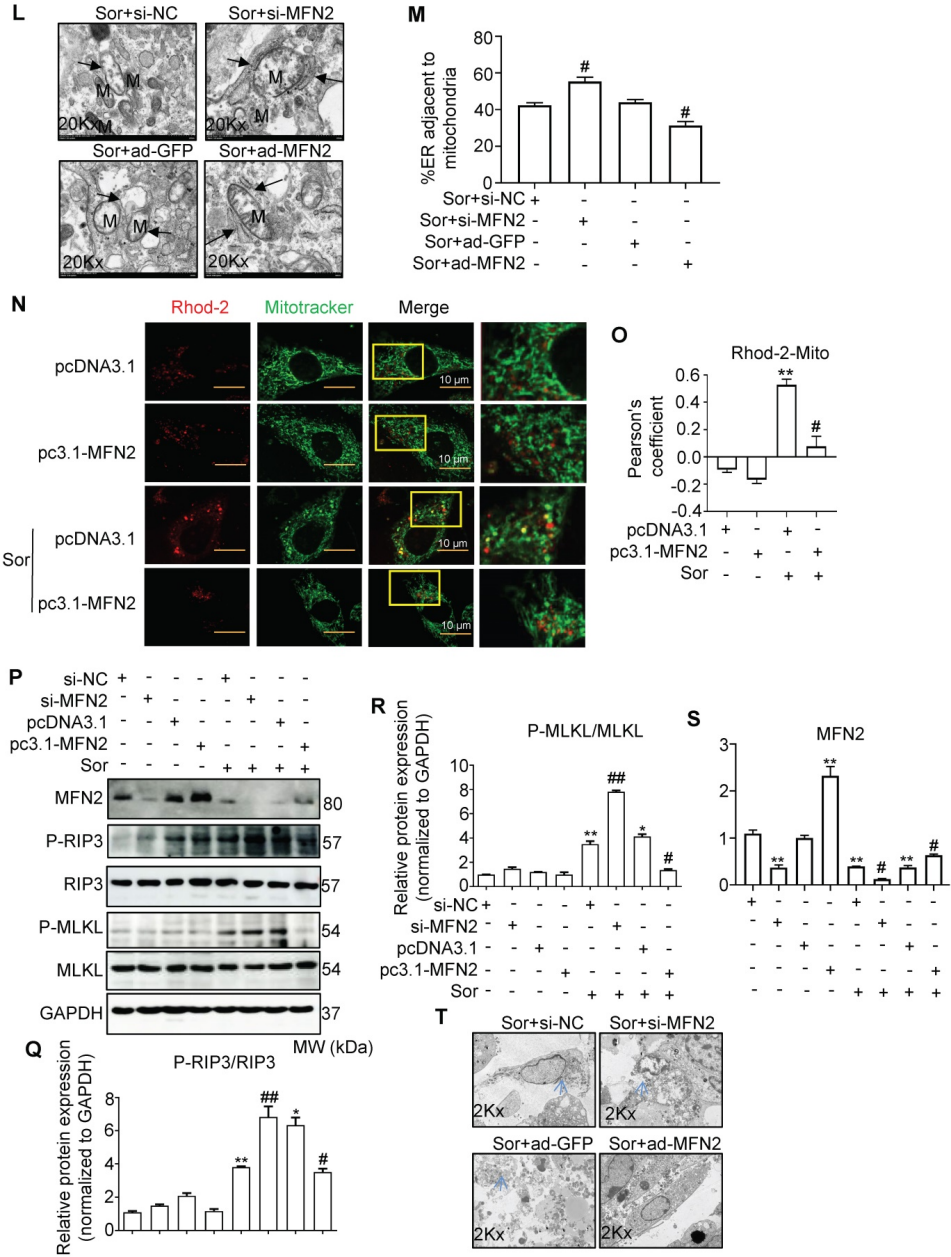
** Downregulation of MFN2 accelerated necroptosis stimulated by sor by regulating ER-mito contact and MAM-component induced Ca2+ overload.** (A, B) Representative blots and quantification of MFN2 protein expression. (C) The relative mRNA level of Mfn2 stimulated with sor, fold change to Gapdh. (D-I) Overexpression or downregulation of MFN2 regulated MAM-derived protein expression and CaMKIIδ expression. (J, K) MFN2 overexpression blocked excessive mitochondria-ER contact, as evaluated by confocal imaging. Scale bar: 10 μm. (L, M) Representative TEM images and quantification of the percent of ER adjacent to mitochondria, under MFN2 overexpression or downregulation. Arrows denote ER. M, mitochondria. (N, O) MFN2 overexpression blocked mitochondrial Ca2+ entry, as detected by confocal imaging. Scale bar: 10 μm. (P-S) Overexpression or downregulation of MFN2 in the regulation of RIP3/MLKL induced necroptosis. (T) Overexpression or down-expression of MFN2 participated in the regulation of cardiomyocytes necrosis shown by TEM. The arrows indicated cell rupture. Sor 20 μM stimulated for 24 h was applied for Figure 4C-T. HL-1 cardiomyocytes were employed for experiments involved in Figure 4J, K, N, O. Primary neonatal mouse cardiomyocytes were employed for experiments involved other legends in Figure 4. ^*^P < 0.05 vs si-NC or ad-GFP or pcDNA3.1. ^**^P < 0.01 vs DMSO or si-NC or ad-GFP or pcDNA3.1. ^#^*P* < 0.05 vs Sor+pcDNA3.1 or Sor+si-NC or Sor+ad-GFP. ^##^*P* < 0.01 vs Sor+pcDNA3.1 or Sor+si-NC or Sor+ad-GFP. N = 3.

To assess mitochondria-MAM tethering, we detected MFN2 expression, a key regulator of MAM, located in the outer mitochondrial membrane (OMM), in cells with or without sor treatment. Western blotting revealed that sor significantly downregulated MFN2 expression in cardiomyocytes in a dose-dependent manner, at the protein level but not at the mRNA level (Figure 4A-C). Overexpression of MFN2 inhibited the expression of MAM component, blocked activated CaMKII and CaMKIIδ protein level in cardiomyocytes (Figure 4D-F). Conversely, silencing of MFN2 increased the expression of MAM component and CaMKIIδ, activated CaMKII in cardiomyocytes (Figure 4G-I). Both confocal and TEM imaging also showed that overexpression of MFN2 blocked mitochondria-MAM contact, while silencing of MFN2 shortened the distance between mitochondria and MAM (Figure 4J-M).

In addition, MFN2 overexpression administration blocked sor-induced excess mitochondrial Ca^2+^ uptake (Figure 4N-O), whereas silence of MFN2 exacerbated sor-induced excess mitochondrial Ca^2+^ uptake (Figure S2C-D). Overexpression of MFN2 significantly inhibited RIP3/MLKL pathway activation. In contrast, silencing of MFN2 had the opposite result (Figure 4P-S). Besides, MFN2 administration blocked sor-mediated cell necrosis shown by TEM images (Figure 4T).

### Overexpression of MFN2 attenuated TSZ-induced cardiomyocyte necroptosis

To confirm whether MFN2 could inhibit cardiomyocyte necroptosis, we employed the classical necroptosis activator, TSZ (composed of TNF-α, SM-164, and Z-VAD-FMK), which triggers necroptosis in cardiomyocytes. Flow cytometry and western blotting analyses revealed that TSZ induced cardiomyocyte necroptosis in a dose-dependent manner (Figure S3A-D). Furthermore, silencing of MFN2 aggravated the TSZ-mediated necroptosis (Figure S3E-F). Conversely, overexpression of MFN2 significantly inhibited TSZ-induced necroptosis (Figure S3G-H).

### Sorafenib promoted mitophagy to degrade MFN2 in the lysosomal ennzyme in cardiomyocytes

**Figure 5:**
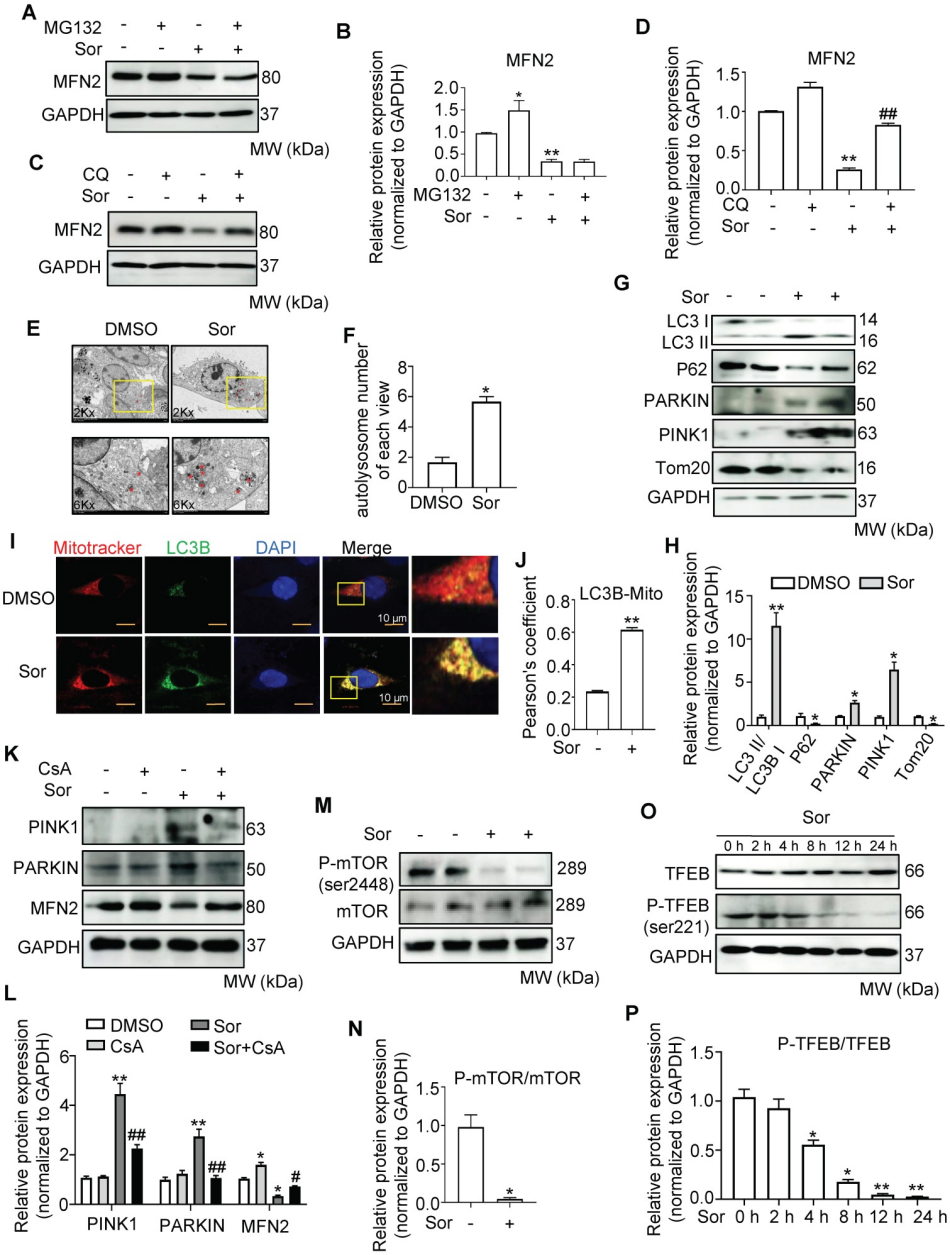

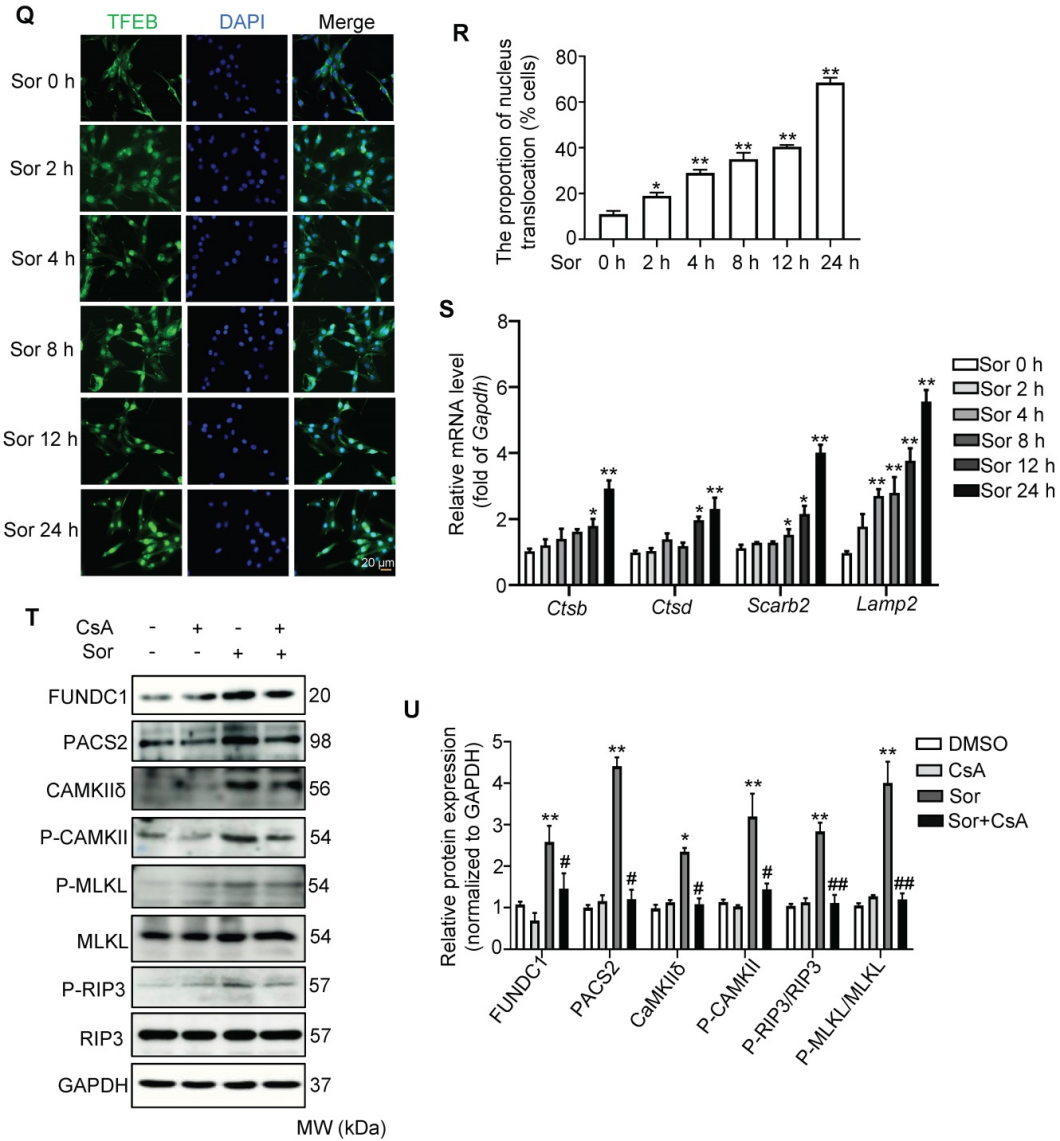
** MFN2 degradation by Sor via lysosomal enzymes was associated with excess mitophagy.** (A-D) Western blotting showed that decreased MFN2 protein level was reversed by CQ, but not MG132. (E, F) Sor triggered excessive autolysosome formation in cardiomyocytes observed by TEM. The red asterisk indicated autolysosome. (G, H) Representative blots and quantification of autophagy-related proteins under sor stimulation. (I, J) MitoTracker and LC3B co-staining was employed to evaluate mitophagy under sor stimulation. Scale bar, 10 μm. (K, L) Representative blots and quantification of proteins of interest under CsA treatment. (M, N) Representative blots and quantification of sor-mediated mTOR activity. (O, P) Representative time-lapse blots and quantification of sor-mediated TFEB activity. (Q, R) Time-lapse of TFEB nuclear translocation after sor stimulation. Scale bar, 20 μm. (S) Time series of mRNA level of lysosomal component under sor stimulation. (T, U) Representative blots to detect necroptosis, MAMs, and CAMKII expression after CsA application and sor stimulation. Sor was employed at 20 μM and HL-1 cardiomyocytes were employed for experiments involved in Figure 5. ^*^P < 0.05 vs DMSO or Sor 0 h. ^**^P < 0.01 vs DMSO or Sor 0 h. ^#^*P* < 0.05 vs Sor. ^##^*P* < 0.01 vs Sor. N = 3.

To explore how sor reduced MFN2 protein levels, we further investigated MFN2 protein levels under MG132 (5 μM) and chloroquine (CQ, 20 μM) treatment 40. Western blotting showed that MG132 enhanced MFN2 expression under physiological conditions, while CQ treatment mainly rescued MFN2 expression in cardiomyocytes with sor stimulation (Figure 5A-D). TEM images showed that sor induced more autolysosome formation compared to DMSO group in cardiomyocytes (Figure 5E-F). Correspondingly, the elevation of LC3B II/I ratios and the reduction of P62 level suggested that autophagy flux was activated. Meanwhile, PARKIN and PINK1 were upregulated, and TOM20 was downregulated by sor stimulation, indicating that mitophagy was also activated (Figure 5G-H). LC3B and MitoTracker co-staining also indicated the occurrence of mitophagy under sor stimulation (Figure 5I-J). Thus the specific mitophagy inhibitor, Cyclosporin A (CsA) was employed to reverse mitophagy induced effects. CsA treatment inhibited the increase of PARKIN and PINK1 and reversed the reduced MFN2 in cells, suggested degradated MFN2 was due to activated mitophagy (Figure 5K-L).

### Sorafenib triggered mitophagy via the mTOR-pTFEB pathway

Sor is an important inhibitor of mTORC1, which regulates autophagy and lysosomal biogenesis via the phosphorylation of TFEB. Next, we detected the expression and activation of mTORC1 and TFEB in cardiomyocytes with or without sor treatment. As expected, sor dramatically inhibited the phosphorylation of mTORC1 at serine 2448 and the phosphorylation of TFEB at serine 211, but did not change the expression of total mTORC1 and TFEB in cells (Figure 5M-P). The increasd TFEB nuclear translocation was observed after treatment with sor for 24 h in a time-dependent manner (Figure 5Q-R). Besides, *Ctsb* (encoded Cathepsin B), *Ctsd* (encoded Cathepsin D), *Lamp2* (encoded LAMP2), and *Scarb2* (encoded LIMP2) mRNA levels dramatically increased after applied with sor in CMs for 24 h in a time-dependent manner (Figure 5S).

To investigate whether mitophagy contributes to sor-induced necroptosis, CsA was used to inhibit excess autophagy induced necroptosis. As shown in Figure 5T and 5U, CsA reversed sor-induced necroptosis via the MAM-CaMKIIδ pathway.

### Sorafenib induced cardiac dysfunction by boosting cardiac necroptosis via regulation of MAMs and CaMKIIδ

**Figure 6:**
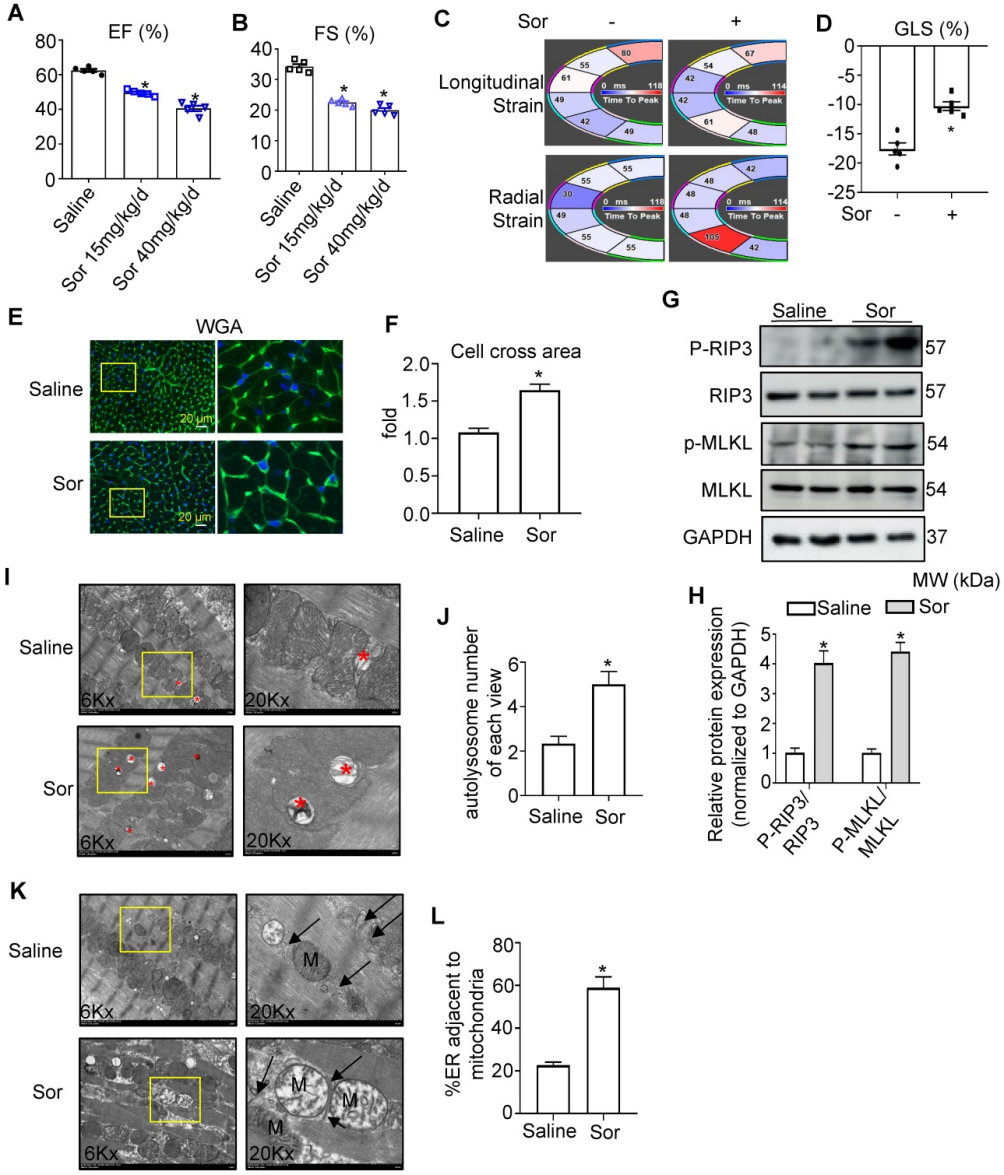
** Sor-induced cardiac dysfunction was associated with MAM-induced necroptosis.** (A, B) Sor-mediated cardiac dysfunction *in vivo* was detected by echocardiography. N = 5. (C, D) Cardiac function was analyzed by Vevo Strain software. N = 5. (E, F) Sor-induced cardiac hypertrophy was detected by WGA staining. Scale bar: 20 μm. (G, H) Sor-induced cardiac necroptosis was investigated by western blotting in cardiac tissue homogenates. N = 3. (I, J) Sor-induced excessive autolysosome formation. The red asterisk indicated autolysosome. N = 5. (K, L) Sor-induced excessive mitochondria-ER contact was evaluated by TEM. N = 5. Arrows denote ER. M, mitochondria. Sor at 40 mg/kg/d for 8 w was employed for Figure 6C-L. ^*^P < 0.05 vs Saline.

To investigate sor-induced myocardial injury *in vivo*, mice were fed with sorafenib by gavage for 8 weeks at a low dose (15 mg/kg/d) and high dose (40 mg/kg/d). Echocardiography and Vevo Strain software analysis showed that sor-mediated cardiac dysfunction, evaluated by EF%, FS%, GLS%, in a dose-dependent manner *in vivo* (Figure 6A-D). Furthermore, Wheat germ agglutinin (WGA) staining revealed cardiomyocyte hypertrophy in sor-treated mice, but not the control group, after 8 w gavage (Figure 6E-F). Phosphorylated RIP3 and Phosphorylated MLKL were significantly upregulated in cardiac tissue homogenates of the sor administration group (Figure 6G-H). Meanwhile, CaMKIIδ expression, CaMKII activation, and MAM-derived protein levels were increased in cardiac homogenates, as confirmed by western blotting (Figure S4A-B). Immunohistochemical staining showed that the expression of PACS2, IP_3_R1, and CaMKIIδ was markedly increased (Figure S4C). The expression of MFN2 was reduced in cardiac tissue, as detected by western blotting and immunohistochemical staining (Figure S4D-F). Besides, excessive autolysosome formation was observed in TEM images (Figure 6I-J). Furthermore, TEM imaging of cardiac slices also showed that sor induced close Mito-ER tethering (Figure 6K-L). Finally, Necrostatin-1 (NEC-1 at 1.65 mg/kg/d for 8 week) reversed sor-induced cardiac necroptosis induced cardiac dysfunction (Figure S5A-D), suggesting that sor indeed induced cardiac necroptosis *in vivo*.

### MFN2 administration reversed sor-induced cardiac dysfunction *in vivo* via the regulation of MAM and CaMKIIδ

**Figure 7:**
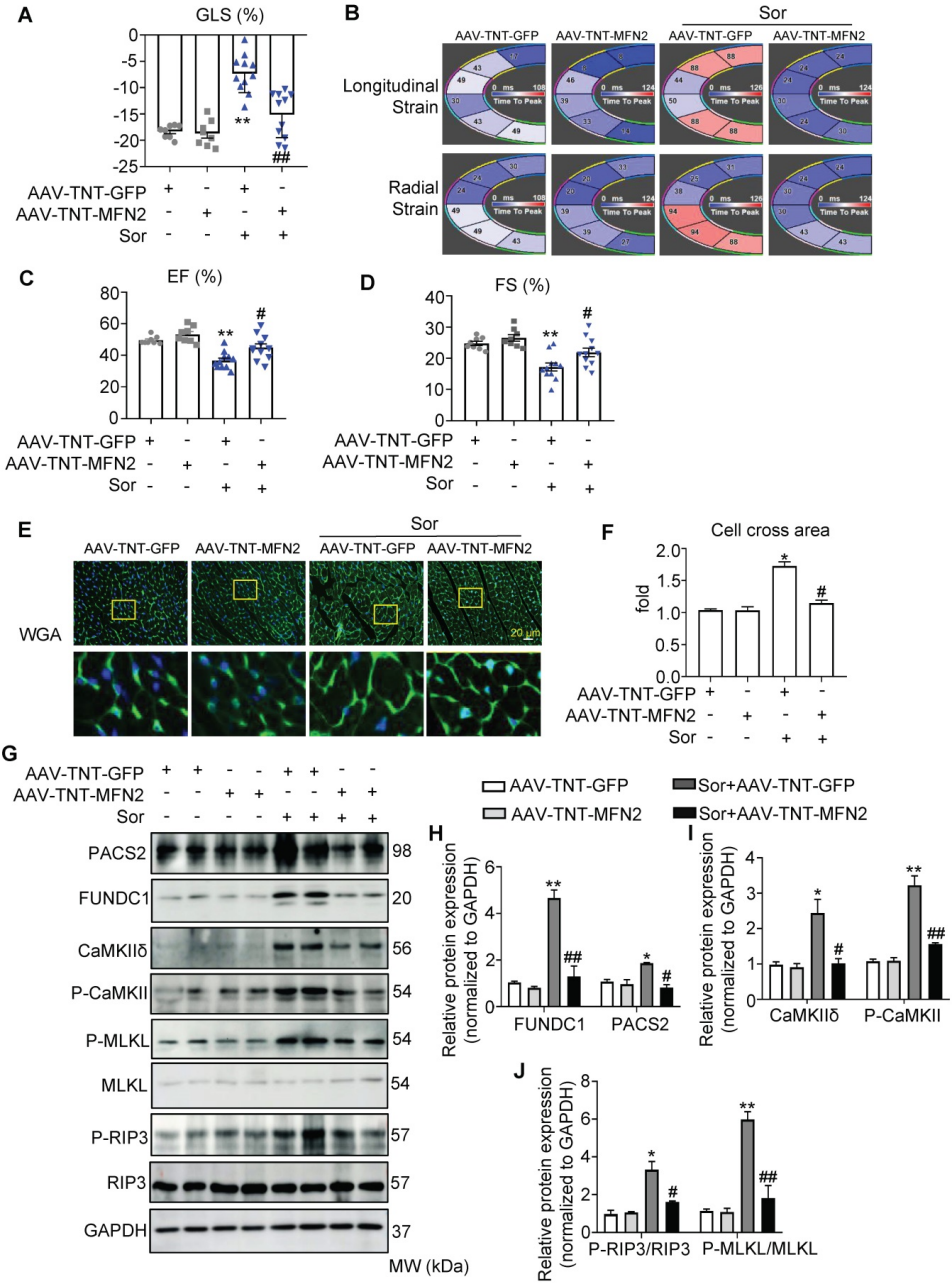
** The cardiac specific overexpression of MFN2 rescued sorafenib-induced cardiac dysfunction *in vivo*.** (A-D) MFN2 overexpression *in vivo* reversed sor-mediated cardiac dysfunction, as measured by echocardiography and analyzed by Vevo Strain software, with parameters including GLS%, EF%, FS%, and Strain. N = 8-11. (E, F) WGA staining to detect cardiac hypertrophy, scale bar, 20 μm. (G-J) MFN2 overexpression *in vivo* reversed MAM-CaMKII-RIP3/MLKL induced necroptosis, as detected in cardiac perfusion protein. N = 3. Sor at 40 mg/kg/d for 8 w was employed for Figure 7. ^*^P < 0.05 vs AAV-TNT-GFP. ^**^P < 0.01 vs AAV-TNT-GFP. ^#^*P* < 0.05 vs Sor+AAV-TNT-GFP. ^##^*P* < 0.01 vs Sor+AAV-TNT-GFP.

Considering that mTOR activators and NEC-1 (a necroptosis inhibitor) cannot be used in cancer therapy, we evaluated whether MFN2 exerted protective effects against sor-induced cardiac injury *in vivo*. As shown in the Figures S6A-I, an adeno-associated virus (AAV) carrying the CMV-GFP-promoter was used to monitor global MFN2 overexpression *in vivo*; global overexpression of MFN2 was found to reverse cardiac dysfunction, cardiomyocyte hypertrophy, and MAM-CaMKII-induced necroptosis. In addition, MAM-mito close-contact was reversed by MFN2 after sor gavage for 8 weeks, as imaged by TEM (Figure S6J-K). Moreover, to better understand the cardiac-specific effect of MFN2 overexpression, an adeno-associated virus carrying a TNT-GFP promoter (AAV-TNT-GFP) was used to monitor MFN2 overexpression *in vivo*. As shown in Figure 7A to 7D, MFN2 overexpression reversed the cardiac dysfunction induced by the 8 weeks administration of sor, as shown by the EF%, FS%, GLS%, longitudinal, and radial strains in the fan diagram. MFN2 administration reversed sor-induced cardiomyocyte hypertrophy (Figure 7E-F). Cardiac perfusion was performed to collect cardiomyocytes to confirm the successful expression of AAV-TNT-GFP-MFN2 in the cardiomyocytes (Figure S7A-C). In addition, western blotting results revealed that MFN2 overexpression inhibited the activation of the MAM-CaMKIIδ pathway as well as subsequent cardiac necroptosis in cardiac perfusion homogenate (Figure 7G-J).

### Sorafenib upregulated the expression of MFN2 in hepatic carcinoma cell line Huh7

MFN2 has been reported to have anti-tumor potential in many types of cancer 14-16, 41, 42. We further investigated whether overexpression of MFN2 affected sor-induced anti-tumor effects. Interestingly, sor treatment increased MFN2 expression in the hepatic carcinoma cell line, Huh7, in a dose-dependent manner (Figure S8A-B). Moreover, knock-down MFN2 using siRNA enhanced PCNA (Proliferating Cell Nuclear Antigen and cell) expression and viability of Huh7 cells and diminished sor-induced necroptosis (Figure S8C-E). Conversely, overexpression of MFN2 inhibited PCNA expression, and increased sor-induced necroptosis in Huh7 cells (Figure S8F-G).

### P53 upregulated MFN2 transcription in Huh7 cells following stimulation with sor

It is obvious that MFN2 was downregulated in cardiomyocytes; however, in contrast, MFN2 was upregulated in Huh7 cells. To clarify the underlying mechanism of this difference, we performed transcriptome chip detection of hepatoma cells and cardiomyocytes with sor stimulation (Figure S9A). Previous studies have reported that P53 status and its mutation are closely related to HCC proliferation and invasion 43, and p53 haploinsufficiency-mediated activation of the PTEN/Akt/mTOR axis promotes HCC tumorigenesis and metastasis, which labels an aggressive subtype of human HCC 44. According to the results of the chip, we found no change in P53 when the cardiomyocytes were stimulated with 20 μM sor (Figure S9B-C); however, in the Huh7 cells, sor stimulated the elevation of *Trp53* (encoded P53) at mRNA level (Figure S9D-F), which was consistent with previous reports 45, 46. Moreover, silence of P53 repressed *Mfn2* expression at the transcriptional level (Figure S9G-I). The above results indicate that P53 played different role in Huh7 cells and cardiomyocytes.

## Discussion

In the present study, we explored sor-triggered cardiomyocyte necroptosis through the MFN2-MAM-Ca^2+^-CaMKIIδ pathway *in vitro* and *in vivo*. Moreover, the overexpression of MFN2 could rescue sor-induced cardiomyocyte necroptosis but did not disturb its anti-tumor effects.

Our results are consistent with a previous study that reported that sorafenib can induce cardiomyocyte necrosis in addition to its anti-tumor effects 7. Therefore, a major problem in the field is the determination of specific cardio-protection targets involved in maintaining or enhancing the effects of anti-tumor medicines. To identify specific cardiac protective targets, we attempted to clarify the mechanism of sor-induced necroptosis in cardiomyocytes. First, we confirmed that the enhancement of mitochondrial Ca^2+^ influx is associated with the activation of CaMKIIδ in sor-induced cardiomyocyte necroptosis. Ca^2+^ overload has been shown to play a crucial role in sor-induced necrosis, both in cardiomyocytes and tumor cells 47-49. CaMKII is a substrate of RIP3 50. As some reports have indicated high levels of CaMKII in various tumor cells, efforts have been made using blockers or inhibitors of CaMKII, of which the most widely studied are KN-93 and KN-62 51. However, one of the limitations of KN-93 and KN-62 is their low potency and the absence of highly specific inhibition. KN-93 and KN-62 cannot discriminate between CaMKII and CaMKIV and also inhibit voltage-gated K^+^ and Ca^2+^ channels 52. Another limitation of KN93 and KN-62 is that these compounds inhibit CaMKII activity by interfering with its binding to the Ca^2+^/CaM complex. However, once CaMKII is activated, its dependence on Ca^2+^/CaM binding is markedly diminished. Thus, these compounds exert considerably less inhibition on CaMKII once it is activated (autophosphorylated) 53.

Furthermore, we found sor induced Ca^2+^ overload in mitochondria by increasing Ca^2+^ import and MCU expression 33, 54. Several studies have shown that MAMs are a key source of mitochondrial Ca^2+^ overload 23, 55. However, the mechanism by which MAMs mediate Ca^2+^ transport to facilitate necroptosis under sor stimulation is unclear. Indeed, our present study confirmed that excessive MAM biogenesis, marked by elevated levels of FUNDC1 and PACS2 and closer between the MAM and mitochondria was stimulated by sorafenib. Silencing of FUNDC1 reversed mitochondrial Ca^2+^ entry, activation of CaMKIIδ, and sor-induced necroptosis, indicating that excess MAM facilitated sor-induced necroptosis. These results confirmed that targeting MAMs may be a potential therapeutic strategy for the treatment of anti-tumor medicine-associated cardiac complications. Furthermore, several studies have reported that FUNDC1 suppresses HCC initiation by blocking inflammatory responses in hepatocytes, whereas upregulation of FUNDC1 expression at the late stage of tumor development may promote tumor growth 56. Moreover, FUNDC1 has been widely reported to be a key receptor for mitophagy 57. Since mitophagy plays a dual role in cancer, whether mitophagy acts as a suppressor or inducer in cancer initiation and development is under debate 58, 59. Further studies are needed to elucidate the role of mitophagy in tumor growth and cancer progression and development, in various circumstances, cell varieties, and cancer types 60.

A major goal for the field is the identification of therapeutic targets involved in MAM regulation. MFN2, located in the OMM, has been widely investigated for its cardiovascular protective effects and is involved in the regulation of MAMs. Under physiological conditions, about 20% of the mitochondrial surface is in close proximity to the ER 61. MFN2, a key fusion protein located both in the mitochondria and ER, can tether the ER and mitochondria to maintain Ca^2+^ transport, lipid and glucose metabolism, which are characterized by MAM 55. The role of MFN2 in maintaining ER-mitochondria (ER-mito) tethering is crucial for Ca^2+^ homeostasis. With the exception of MFN2, a series of different proteins have been reported to play a role in regulating the ER-mito juxtaposition, such as IP3R, VDAC1, GRP75, PACS2, DRP1, and MFN1 62. A previous study demonstrated that MFN2-KD-induced repression of mitochondrial Ca^2+^ uptake occurred as a result of downregulated MCU, which modulated the mitochondrial Ca^2+^ transport machinery 21. Controversially, another study reported that the acute deletion of MFN2 by siRNA promoted ER and mitochondria coupling (clearly demonstrated in the study and shown by TEM images 21 ), which enhances ER-mito contact to accelerate Ca^2+^ release from the ER and transport into the mitochondria. In our study, we demonstrated that sor stimulation could elevate the expression of MAM proteins, such as FUNDC1 and PACS2, thus boosting the biogenesis of MAM as well as ER-mito tethering. Furthermore, downregulation of MFN2 at the protein level, mediated by sor, further aggravated the sor-induced expression of the MAM proteins and tethering between the ER and mitochondria. Specifically, some significant evidence has revealed that the difference in MFN2-mediated SR-mitochondrial communication depends on mitochondrial location and type of communication (IP3R-mRyR1 vs. ryanodine receptor type 2-mitochondrial calcium uniporter) or the stimulation form 63. In conclusion, it is important to understand the impact of MFN2 deficiency/overexpression on Ca^2+^ uptake and ER-mito contact in a comprehensive manner, in terms of the pathological stimulation and mitochondrial location, MAM biogenesis, etc.

Furthermore, some studies have reported that MFN2 exerts anti-tumor effects; in our study, we also confirmed that MFN2 promoted RIP3/MLKL complex-induced necroptosis in the Huh7 cell line. In the current study, after treatment with sorafenib, MFN2 expression was decreased in cardiomyocytes but increased in the hepatoma cell line. Correspondingly, MFN2 reversed necroptosis in cardiomyocytes but promoted sor-induced necroptosis in Huh7 cells. These results indicated that MFN2 not only showed cardioprotective effects, but also augmented the sor-induced anti-tumor effect. According to previous literature reports and our study, when normal cells undergo malignant transformation into tumor cells, a variety of signaling molecules in the cells undergo change. For example, the activation of many critical pathways, including Ras/Raf/MAPK, PI3K/AKT/mTOR, Wnt/β-catenin signaling pathways, ubiquitin/proteasome degradation, and the hedgehog signaling pathway, might lead to novel therapeutics for HCC treatment 64. The use of sor, a multi-kinase inhibitor that blocks classical kinases such as MAPK, PDGF, VEGF, may benefit anti-tumor therapy 65. In contrast, in normal cells, the multiple kinase pathways mentioned above are maintained in an inactive state, and thus will not respond to sor intervention. Similarly, in Huh7 cells, we detected that the expression of MFN2 was increased at the mRNA level, which is regulated by P53 (shown in Figure S9). However, in normal cardiomyocytes, we found that the expression of MFN2 induced by sor intervention was reduced at the protein level, but not at the mRNA level. This may be related to the repression of mTOR and activation of TFEB, dephosphorylation-induced TFEB nuclear translocation and activated mitophagy. In the current study, CsA administration inhibited the fusion of autophagosomes and lysosomes, and reversed excess mitophagy and the subsequent MAM-Ca^2+^-CaMKII-induced necroptosis. As MFN2 exerts benefits for both the heart as well as for anti-cancer therapies, MFN2 is a potential therapeutic target for clinical translation.

There are still some additional limitations to our study. Some other potential molecules besides MFN2 may benefit both tumor therapy and tumor-associated cardiac dysfunction. Furthermore, due to the lack of clinical studies with MFN2 intervention in various patient cohorts, whether MFN2 can benefit tumor patients with cardiac complications still needs further study.

## Conclusions

Altogether, we confirmed that sor-mediated excess MAM-derived mitochondrial Ca^2+^ entry and the subsequent activation of CaMKII contributed to necroptosis in cardiomyocytes, and can be repressed by MFN2 via regulation of MAMs and CaMKIIδ overproduction as well as the blocking of mitochondrial Ca^2+^ overload. We suggest that MFN2 is a promising target for the treatment of anti-cancer medicine-induced cardiac dysfunction.

## Supplementary Material

Supplementary methods, figures and tables.

## Abbreviations

MFN2: mitofusin-2
CaMKIIδ: calmodulin-dependent protein kinase II delta
MAM: mitochondria-associated endoplasmic reticulum (ER) membrane
TEM: transmission electron microscopy
RIPK1: receptor-interacting serine/threonine-protein kinase 1
RIPK3: receptor-interacting serine/threonine-protein kinase 3
MLKL: mixed lineage kinase domain-like protein
mTOR: mammalian Target of Rapamycin
TFEB: transcription factor EB
VEGF: vascular endothelial growth factor
MAPK: mitogen-activated protein kinase
HCC: hepatocellular carcinoma
TNFR: tumor necrosis factor receptor
FasR: Fas ligand receptor
TLRs: toll-like receptors
mPTPs: mitochondrial permeability transition pores
DAMPs: damage-associated molecular patterns
MCU: mitochondrial Ca^2+^ uniporter
OMM: outer mitochondrial membrane
CQ: chloroquine
WGA: wheat germ agglutinin
AAV: adeno-associated virus
PCNA: Proliferating Cell Nuclear Antigen
PDGF: platelet-derived growth factor
CMs: cardiomyocytes
