# Supplementary material for: Overexpression of MFN2 alleviates sorafenib-induced cardiomyocyte necroptosis via the MAM-CaMKIIδ pathway in vitro and in vivo
Source: Theranostics. 2022 Jan 1;12(3):1267–85. doi: 10.7150/thno.65716 (PMC8771548; doi:10.7150/thno.65716)
Supplement: Supplementary file 1 — Supplementary methods, figures and tables. [file thnov12p1267s1.pdf]

Supplemental Materials

Supplemental Methods:

Autophagy detection by LC3B-Mitotracker co-staining

Briefly, cardiomyocytes were transfected with adenovirus loaded EGFP-LC3B, after various treatment, cells were co-stained with MitoTracker™ Deep Red FM (M22426, Invitrogen, Thermo Fisher Scientific Inc.), then cells were fixed and imaged by confocal microscope, and analyzed using the Zeiss colocalization module in the ZEN software.

Immunofluorescence staining

After cardiomyocytes were subjected to various treatments, they were rinsed with PBS once and fixed for 15 min with 4% paraformaldehyde in PBS. The slides were washed twice with PBS and cells were permeabilized with 0.02% Triton X-100 for 10 min. After rinsing twice with PBS, the slides were incubated with primary antibody of TFEB in a 1:100 dilution at 4°C overnight. Next day, slices were incubated with secondary antibodies for 40 min at room temperature in the dark, washed three times with PBS followed by DAPI co-staining. Slides were mounted on glass coverslips and imaged by fluorescence microscope.

Cell area measurement

Wheat germ agglutinin (WGA) staining was employed for cardiac tissue slices for detection of cardiomyocytes size. WGA dye was diluted in water firstly, and slices were incubated with WGA work solution at 37°C for 30 min. Then, DAPI was co-stained for 5 min, and slices were observed and imaged by fluorescence microscope.

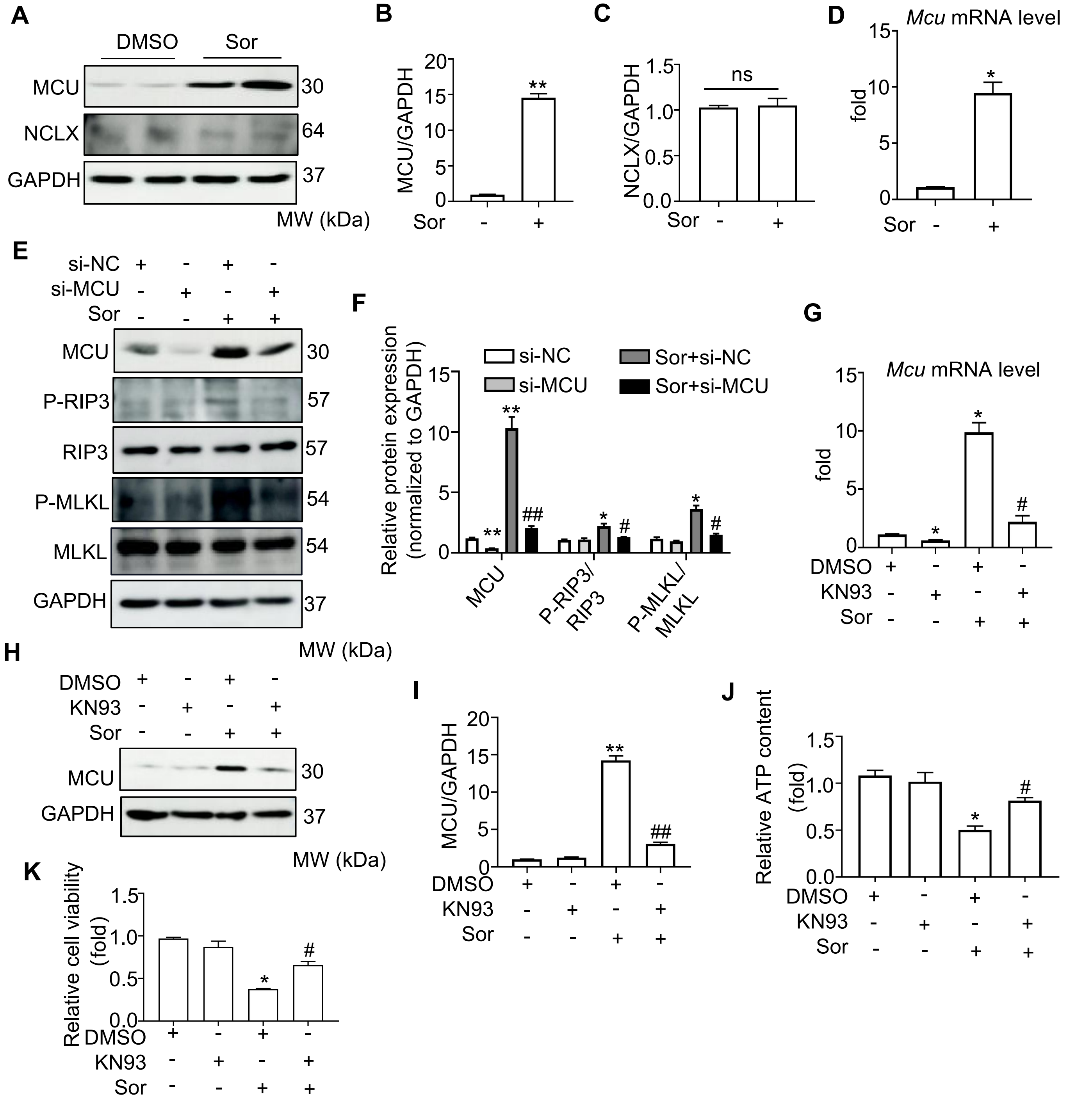

Figure S1. Mitochondrial Ca^{2+} channel protein, MCU was upregulated due to increased CaMKII activity under Sor stimulation. (A-C) Representative blots and analysis of proteins of interest related to mitochondrial Ca^{2+} entry and export. (D) The mRNA levels of MCU with Sor stimulation. (E, F) Western blot imaging and analysis of necroptosis under Sor and MCU siRNA stimulation. (G-I) The mRNA and protein levels of MCU with or without Sor and KN93 intervention. (J, K) ATP content and cell viability were detected under Sor stimulation. Sor treatment (20 μM) for 24 h was employed for Figure S1. $^{*}P$ < 0.05 vs DMSO or si-NC. $^{**}P$ < 0.01 vs DMSO or si-NC. $^{\#}P$ < 0.05 vs Sor or Sor+si-NC. $^{\#\#}P$ < 0.01 vs Sor or Sor+si-NC. N = 3.

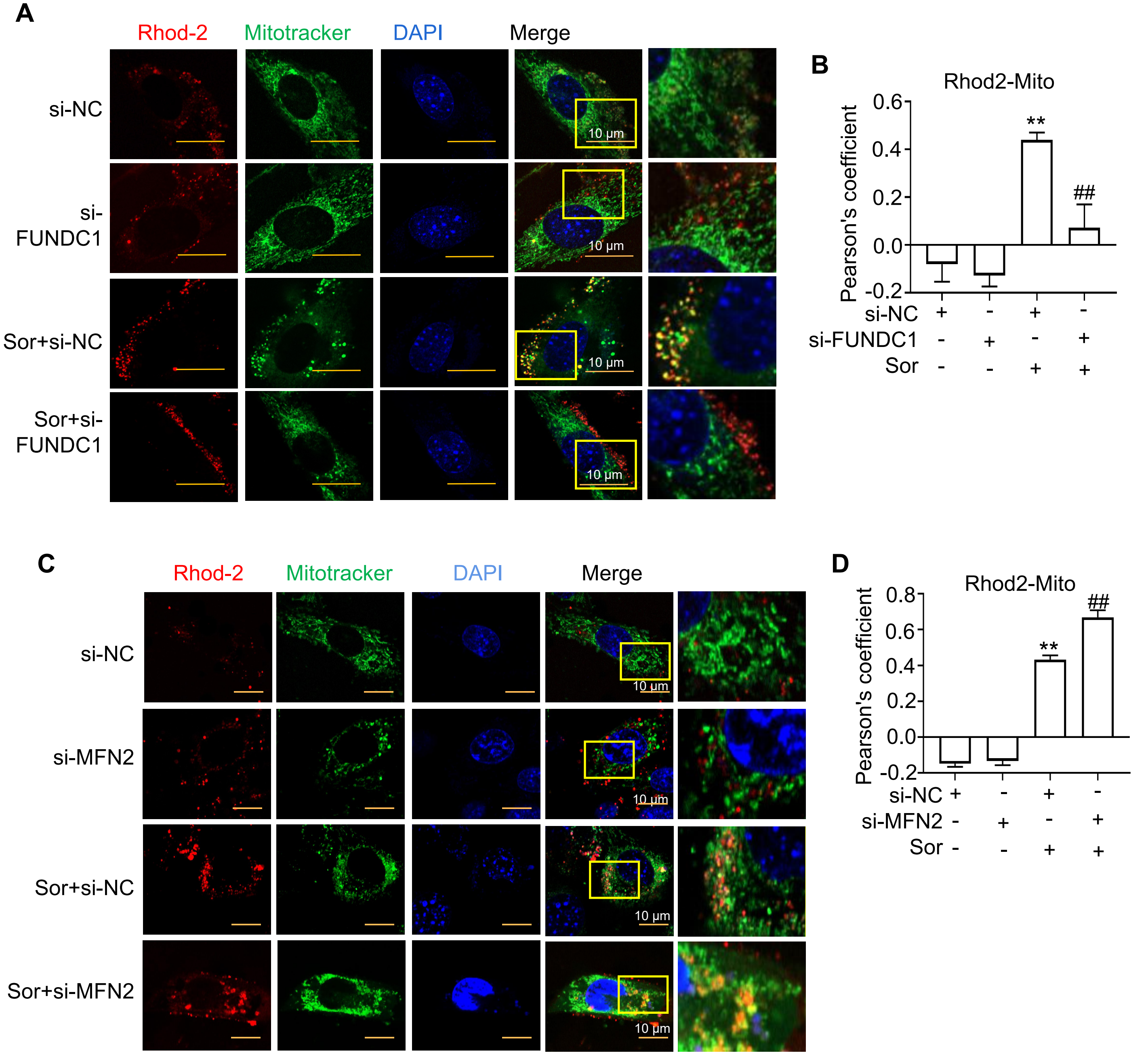

Figure S2. Silencing of FUNDC1 and silencing of MFN2 repressed Sor-induced excess Ca^{2+} entry. (A, B) Rhod2 and MitoTracker co-staining was performed to detect Ca^{2+} change with si-FUNDC1 treatment. Quantitative analysis of Pearson's coefficient was performed. (C, D) Rhod2 and MitoTracker co-staining was performed to detect Ca^{2+} change with si-MFN2 treatment. Quantitative analysis of Pearson's coefficient was performed. Scale bar: 10 μm. $^{**}P < 0.01$ vs si-NC. $^{\#\#}P < 0.01$ vs Sor+si-NC. N = 4.

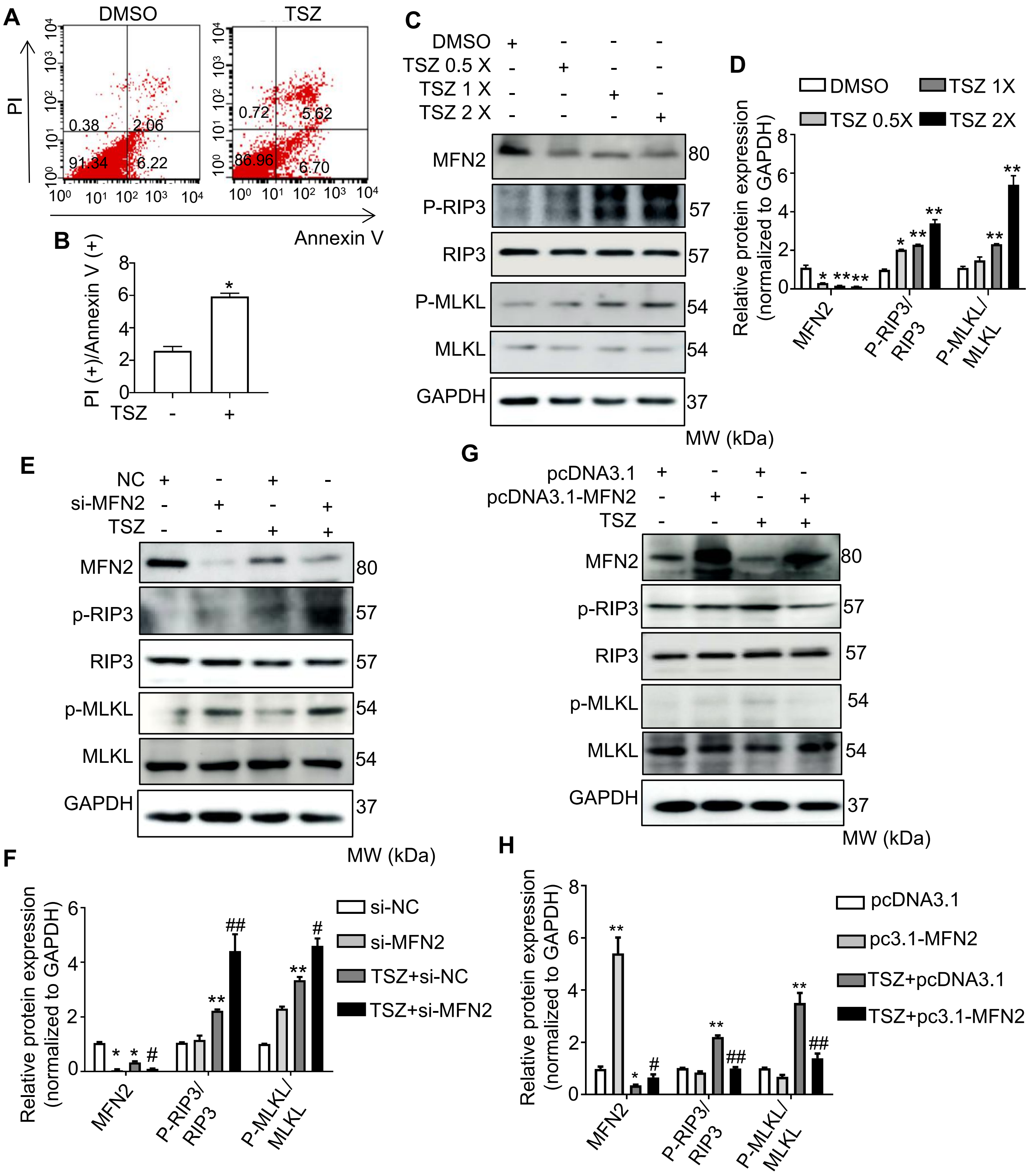

Figure S3. MFN2 repressed TSZ-induced necroptosis in cardiomyocytes. (A, B) TSZ-induced cell necroptosis was evaluated by flow cytometry. (C, D) Representative blots and quantification of the necroptosis pathway with TSZ applied under a concentration gradient. (E, F) Representative blots and quantification of the regulation of the necroptosis pathway mediated by MFN2 overexpression, under TSZ stimulation. (G, H) The effect of the down-regulation of MFN2 in the regulation of TSZ-induced necroptosis. 1X TSZ was applied for Figure S3E-H. *P < 0.05 vs DMSO or si-NC or pcDNA3.1. **P < 0.01 vs DMSO or si-NC or pcDNA3.1. #P < 0.05 vs Sor+si-NC or pcDNA3.1. ##P < 0.01 vs Sor+si-NC or pcDNA3.1. N = 3.

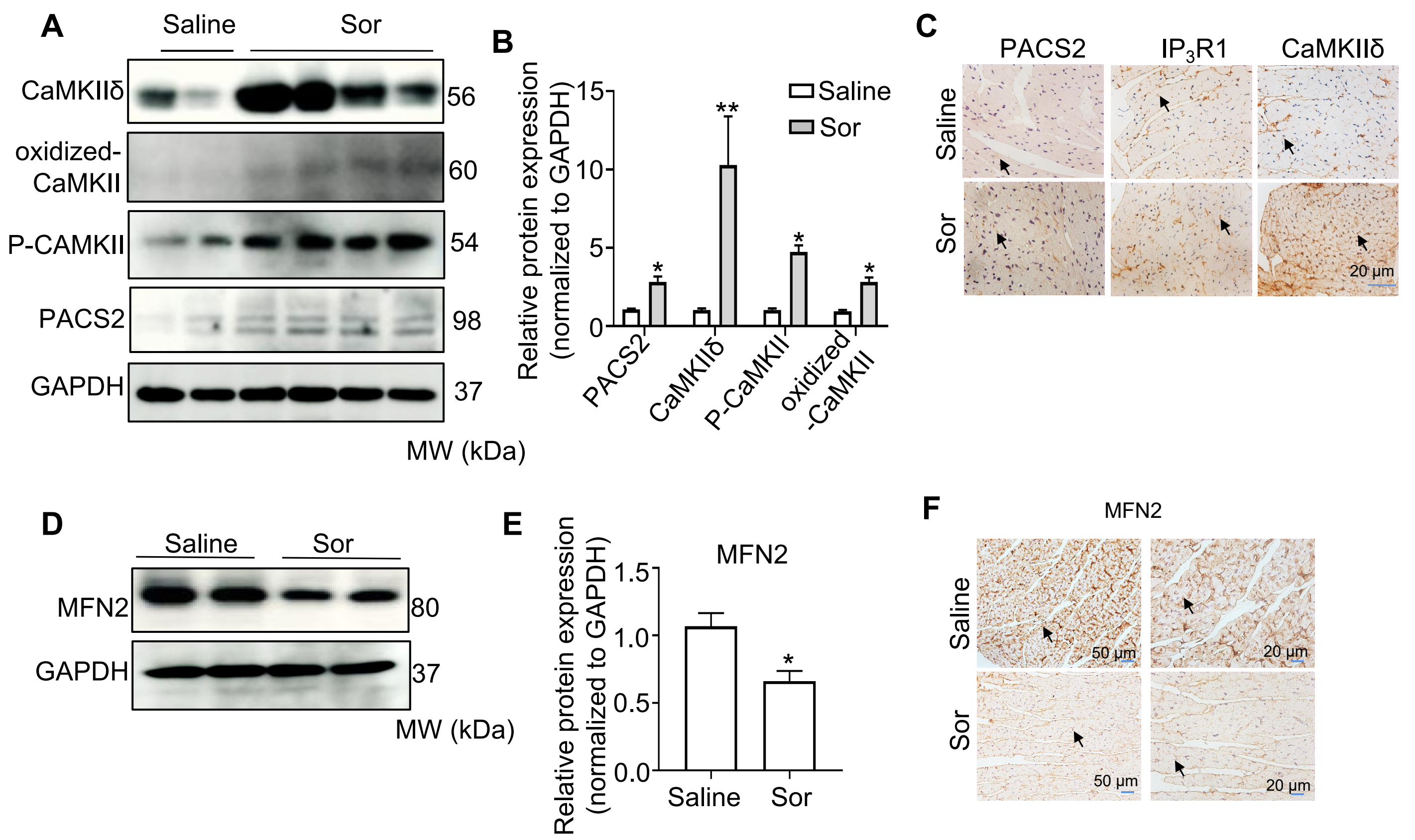

Figure S4. Sor induced CaMKII activation and MAM overproduction *in vivo*. (A, B) Representative blots and quantification of proteins of interest in heart under Sor gavage at 40mg/kg/d for 8 weeks. (C) Immunohistochemical staining of MAM-derived protein. (D, E) Representative blots and quantification of interest proteins in cardiac homogenate. (F) Immunohistochemical staining of MFN2 in cardiac slices. *$P < 0.05$ vs Saline. **$P < 0.01$ vs Saline. N = 3.

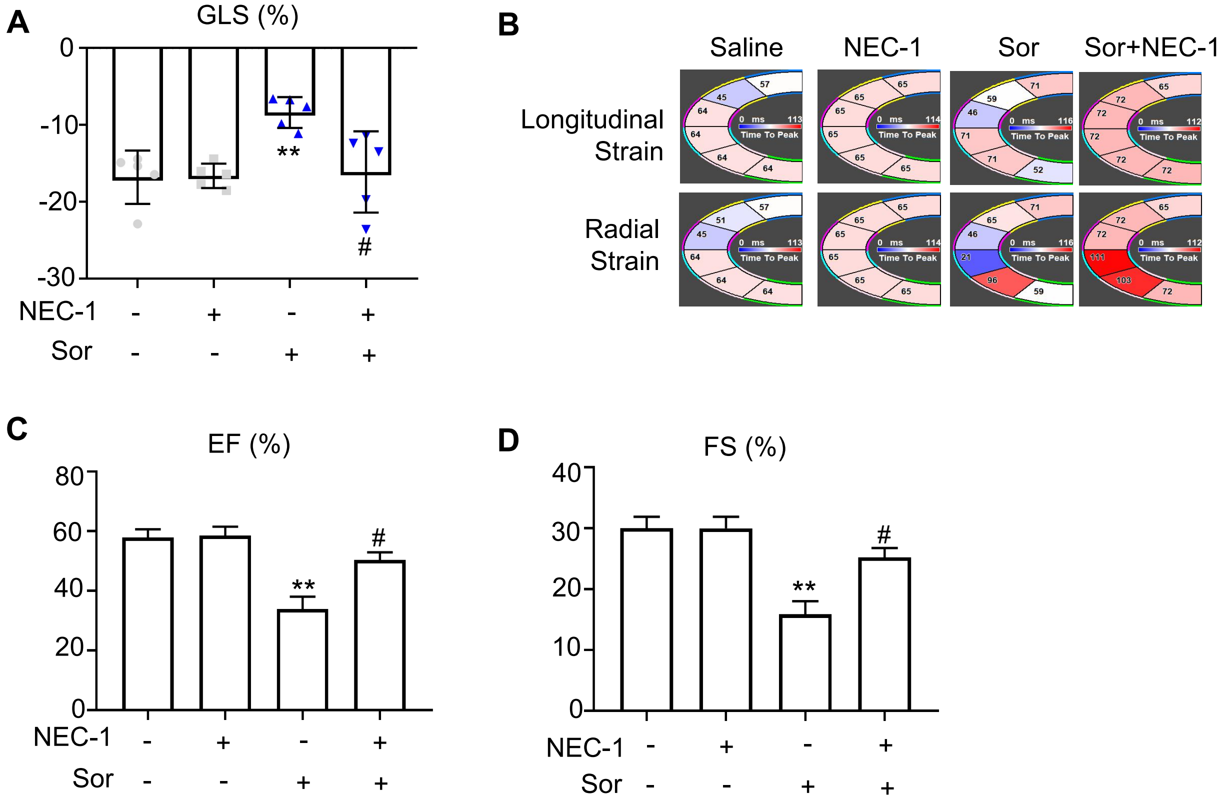

Figure S5. NEC-1 rescued Sor-mediated cardiac dysfunction *in vivo*. (A-D) Echocardiography of mice given NEC-1 subcutaneously at 1.65 mg/kg/d for 8 weeks. Analysis was performed by Vevo Strain software, with parameters including GLS%, cardiac longitudinal and radial strain, EF%, FS%. **$P < 0.01$ vs Saline. #$P < 0.05$ vs Sor. N = 5.

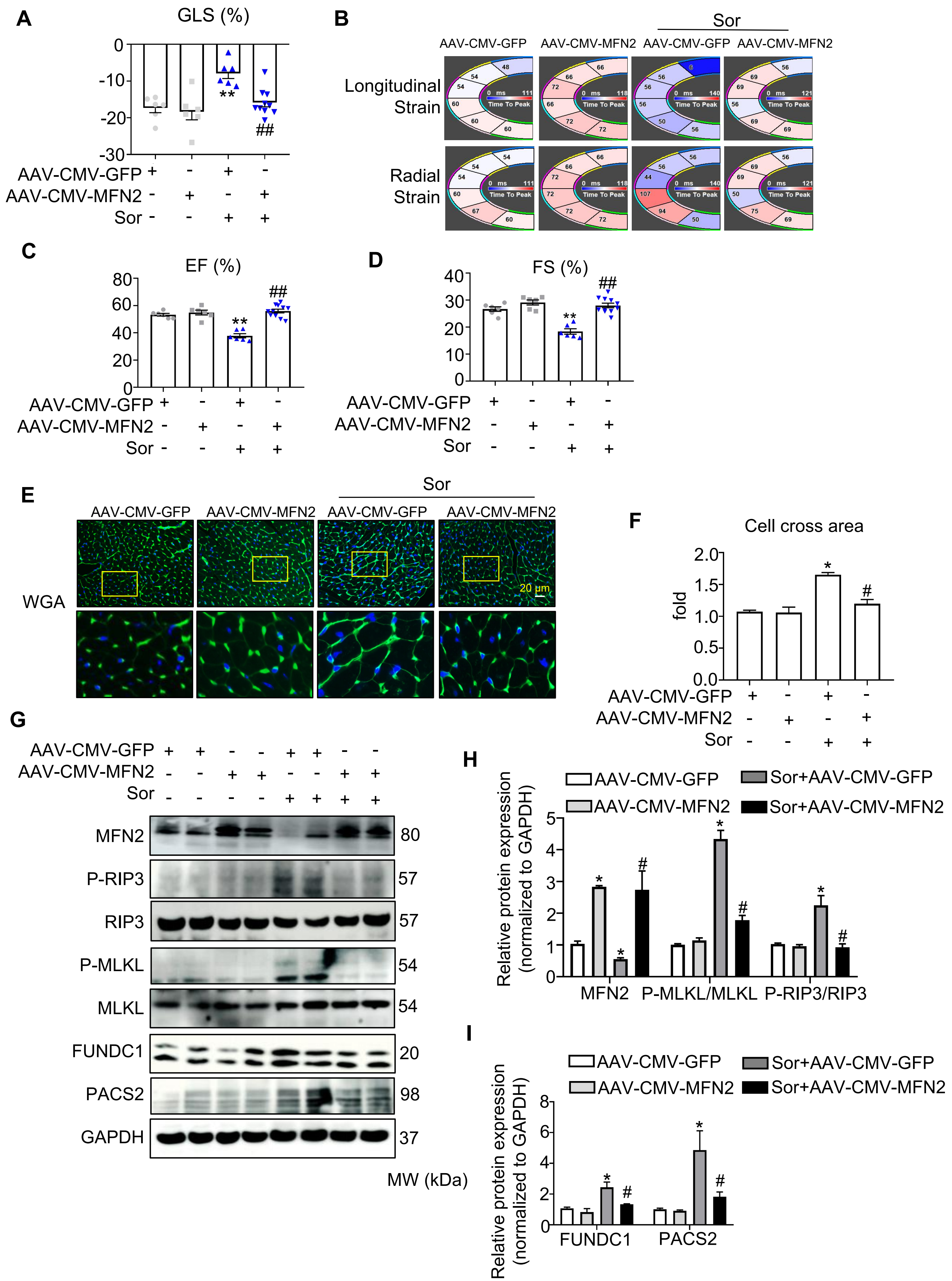

Figure S6. The global overexpression of MFN2 rescued Sorafenib-induced cardiac dysfunction in vivo by restraining excessive RIP3/MLKL pathway activation. (A-D) MFN2 overexpression in vivo reversed Sor-mediated cardiac dysfunction after 8 weeks Sor gavage, as measured by echocardiography and analyzed by Vevo Strain software, with parameters including GLS%, EF%, FS%, and Strain. N=6-10. (E-F) WGA staining showed that MFN2 reversed Sor-induced cardiomyocyte hypertrophy. Scale bar: 20 μm. (G-I) MFN2 overexpression in vivo reversed myocardial necroptosis and MAM expression after 8 weeks Sor gavage, as detected in cardiac tissue homogenates. N = 3.

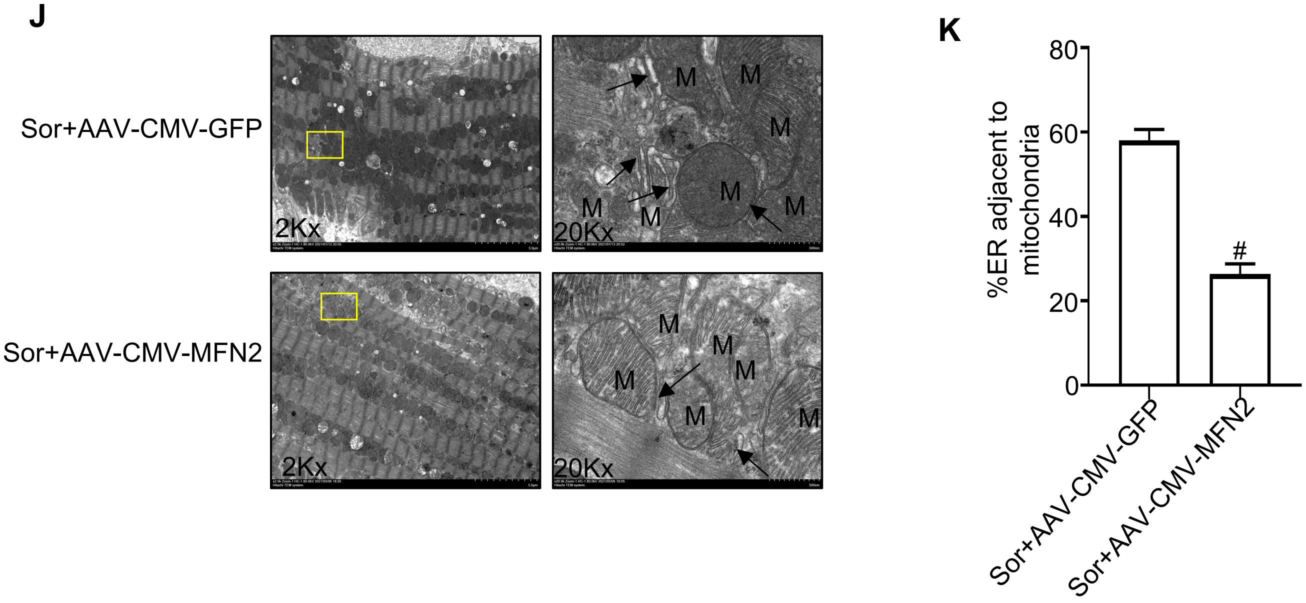

(J, K) The morphology of mito-ER contacts with Sor intervention was imaged by TEM. Arrows denote ER. M, mitochondria. Sor at 40mg/kg/d for 8 w was employed for Figure S6. $^{*}P < 0.05$ AAV-CMV-GFP. $^{**}P < 0.01$ vs AAV-CMV-GFP. $^{\#}P < 0.05$ vs Sor+AAV-CMV-GFP. $^{\#\#}P < 0.01$vs Sor+AAV-CMV-GFP.

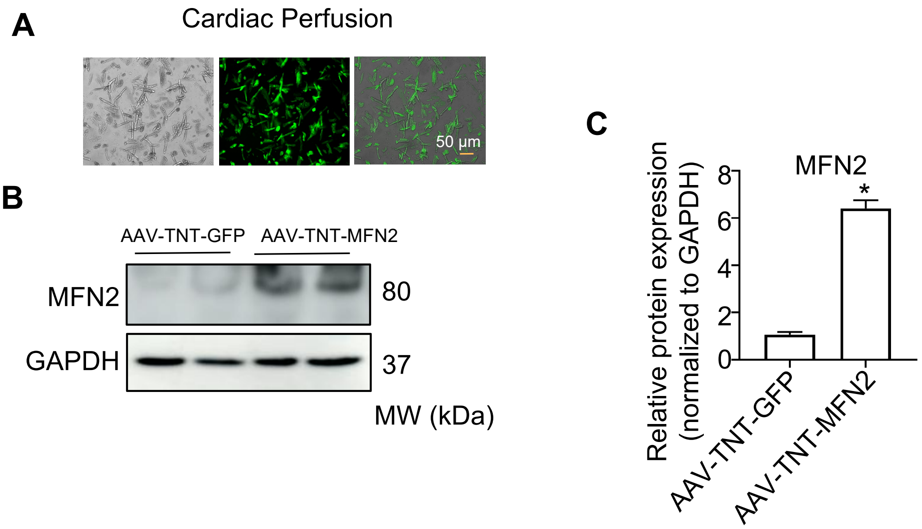

Figure S7. Overexpression of AAV-TNT-MFN2 in vivo. (A, B) Morphology of cardiomyocytes collected by cardiac perfusion after 8 weeks of Sor application. (C) Successful delivery of AAV-GFP-TNT, as shown by imaging. $^{*}P < 0.05$ vs AAV-TNT-GFP. N = 4.

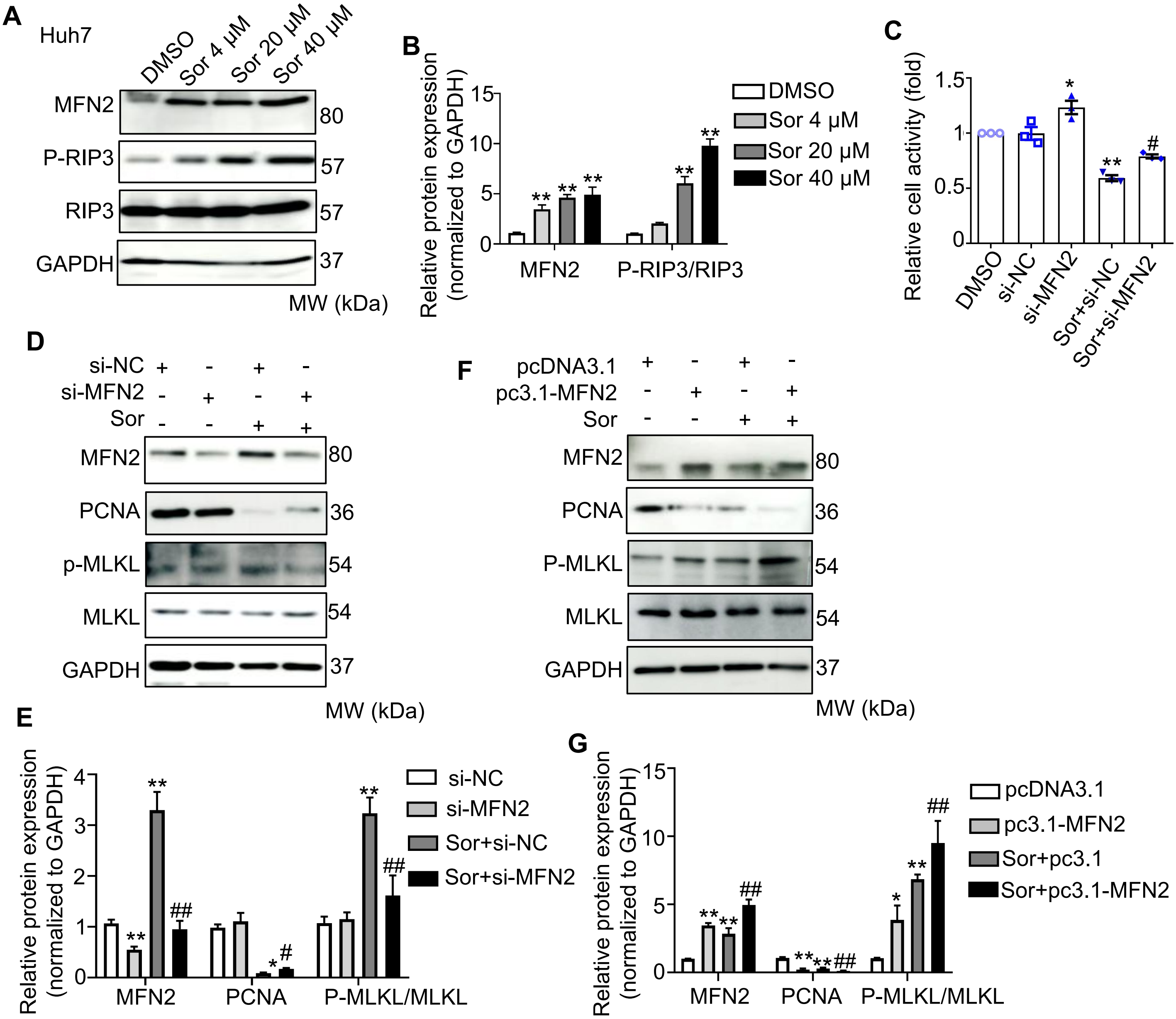

Figure S8. MFN2 was upregulated in Huh7 cells and regulated hepatoma cell proliferation and viability. (A, B) Representative protein expression and quantification of MFN2 level and necroptosis in Huh7 cells. (C) MFN2 regulated cell viability in Huh7 cells under sor stimulation, as detected by CCK8 assays. (D-G) The effect of the overexpression and down-regulation of MFN2 on the regulation of hepatoma cell proliferation and necroptosis. $^{*}P < 0.05$ vs DMSO or si-NC. $^{**}P < 0.01$ vs DMSO or si-NC or pcDNA3.1. $^{\#}P < 0.05$ vs Sor+si-NC. $^{\#\#}P < 0.01$ vs Sor+si-NC or Sor+pc3.1. N = 3.

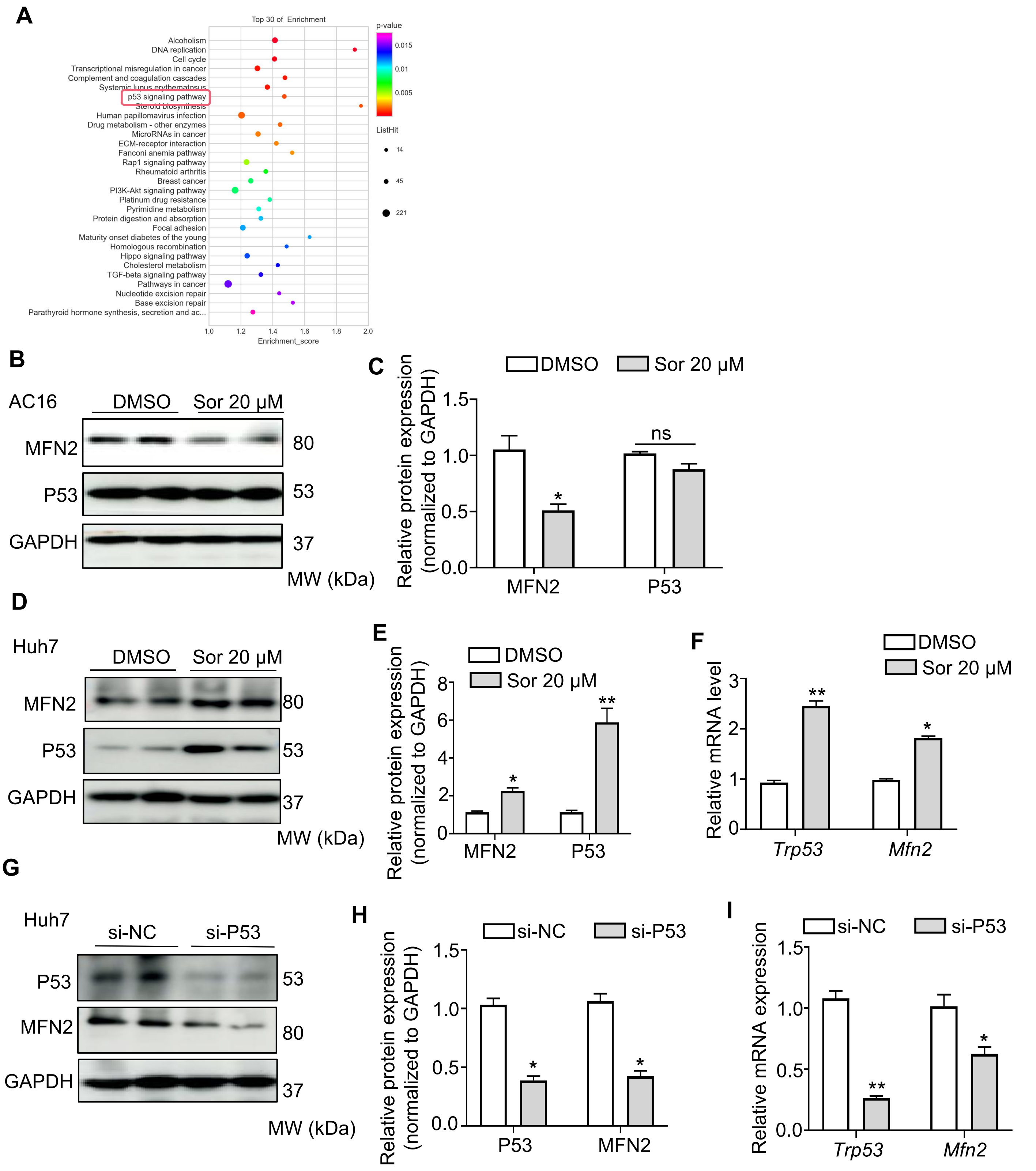

Figure S9. P53 upregulated MFN2 at the transcriptional level in Huh7 cells stimulated with Sor. (A) Pathway analysis of AC16 and Huh7 cell lines under Sor stimulation. (B, C) Representative blots and quantification of p53 and MFN2 in AC16 cells. (D, E) Representative blots and quantification of P53 and MFN2 in Huh7 cells. (F) Relative mRNA levels of P53 and MFN2 in Huh7 cells under Sor application. (G-I) Representative blots and quantification and relative mRNA levels of P53 and MFN2 under si-P53 application in Huh7 cells. $^{*}P < 0.05$ vs.vs DMSO or si-NC. $^{**}P < 0.01$ vs DMSO or si-NC. N = 3.

Table S1

primers in our study

Gene	Forward primers	Reverse primers
m-*Camk2d*	GATGGGGTAAAGGAGTCAACTG	CATTGTGGCATACAGCGACA
m-*Gapdh*	AGGTCGGTGTGAACGGATTTG	TGTAGACCATGTAGTTGAGGTCA
m-*Scarb2*	AGAAGGCGGTAGACCAGAC	GTAGGGGGATTTCTCCTTGGA
m-*Lamp2*	TGTATTTGGCTAATGGCTCAGC	TATGGGCACAAGGAAGTTGTC
m-*Ctsd*	GCTTCCGGTCTTTGACAACCT	CACCAAGCATTAGTTCTCCTCC
m-*Ctsb*	TCCTTGATCCTTCTTTCTTGCC	ACAGTGCCACACAGCTTCTTC
m-*Fundc1*	CCCCCTCCCCAAGACTATGAA	CCACCCATTACAATCTGAGTAGC
m-*Pacs2*	GCAGGAAGCGGTACAAGAACA	CTGGACAGGGAGACGATCC
m-*Mfn2*	AGAACTGGACCCGGTTACCA	CACTTCGCTGATACCCCTGA
m-*Mcu*	GAGCCGCATATTGCAGTACG	CGAGAGGGTAGCCTCACAGAT

Table S2

Primary antibodies involved in our study

Antibodies	Company	Catalog number
MFN2	Cell Signaling Technology	#9482
Phospho-MLKL-T357/S358/S360	ABclonal	AP0949
MLKL	ABclonal	A5579
Phospho-RIP3 (Thr231/Ser232)	Cell Signaling Technology	91702s
RIP3	ABclonal	A5431
CaMKIIδ	Abcam	ab181052
oxidized-CaMKII (Met281/282)	Sigma	07-1387
Phospho-CaMKII alpha (Thr286)	Thermo Fisher	MA1-047
PACS2	Proteintech	19508-1-AP
FUNDC1	ABclonal	A16318
GAPDH	Cell Signaling Technology	#5174
MCU	Cell Signaling Technology	#14997
NCLX	Abcam	ab83551
Phospho-mTOR (Ser2448)	Cell Signaling Technology	#5536
mTOR	Cell Signaling Technology	#2983
PARKIN	Cell Signaling Technology	#2132
PINK1	Abcam	ab216144
LC3B	Cell Signaling Technology	#43566
P62	Cell Signaling Technology	#48768
TFEB	Cell Signaling Technology	#32361
Phospho-TFEB (Ser211)	Cell Signaling Technology	#37681
TOM20	Cell Signaling Technology	#42406
